# Supplementary figures and images for: A spatially resolved stochastic model reveals the role of supercoiling in transcription regulation
Source: PLoS Comput Biol. 2022 Sep 19;18(9):e1009788. doi: 10.1371/journal.pcbi.1009788 (PMC9522292; doi:10.1371/journal.pcbi.1009788)

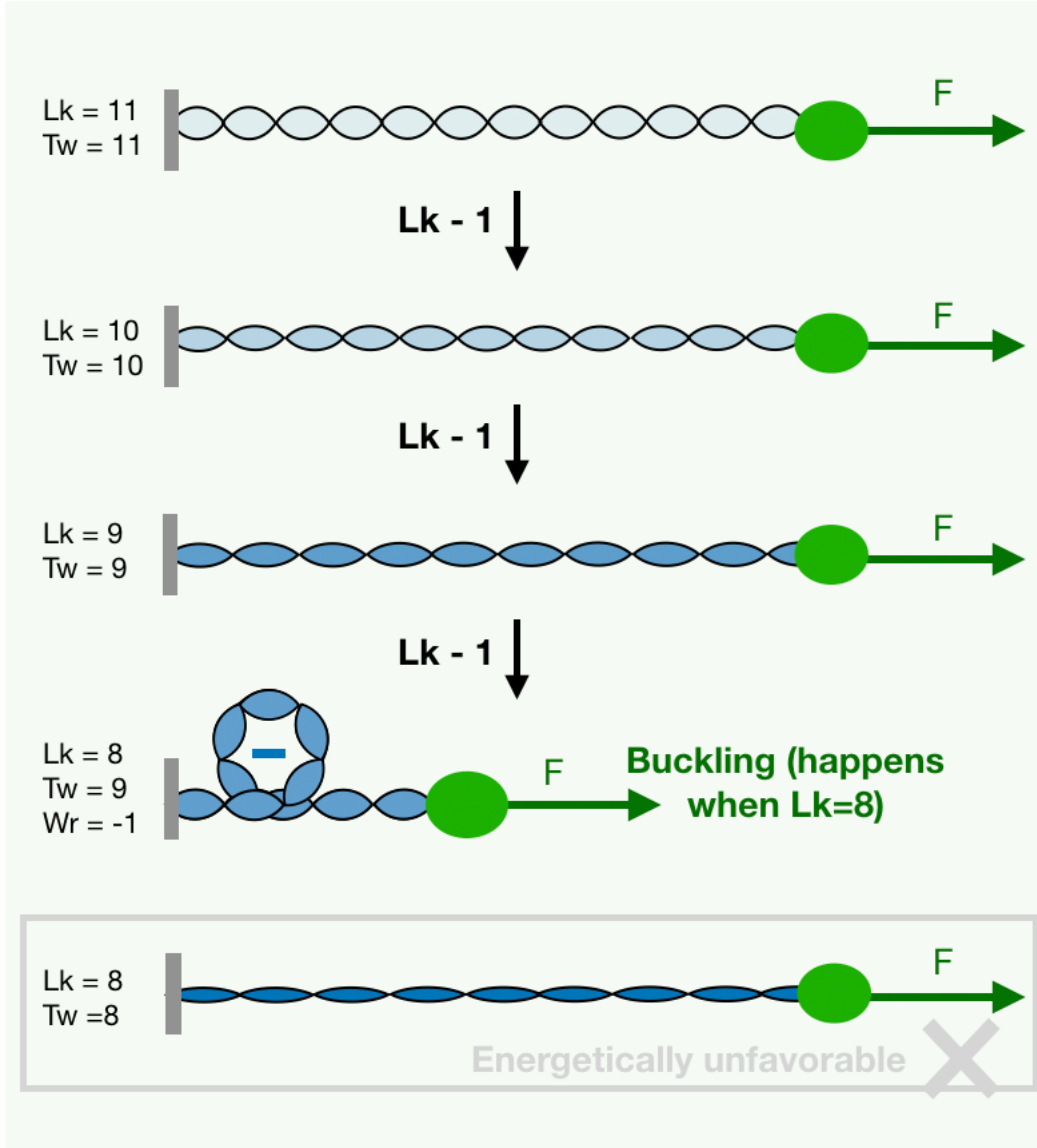

Supplement: S1 Fig — The left end of DNA is fixed on a topological barrier, and the right end is pulled by force F. Initially, DNA exists in the form of pure twists. We reduce the linking number by one at each step (and it could be removed by topoisomerase or other mechanisms). At first, DNA reduces the density of twists. When the linking number is reduced from 9 to 8, the DNA buckles itself to form a negative supercoil and preserves the number of twists in the previous step. (PDF) [file pcbi.1009788.s001.pdf]

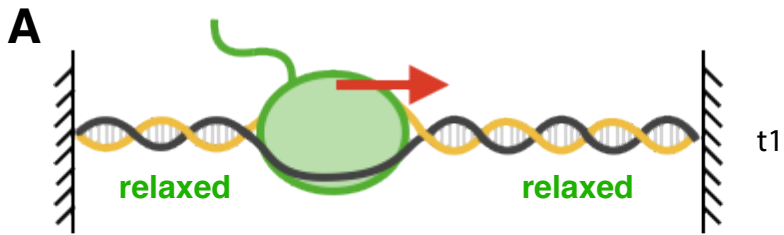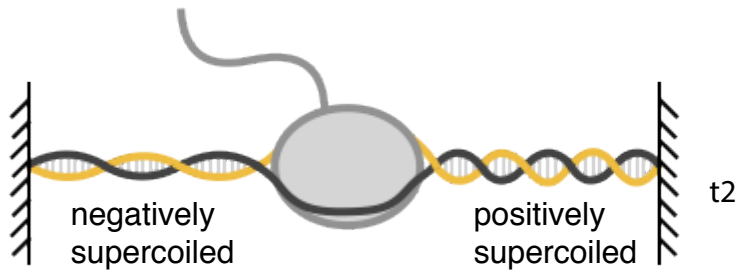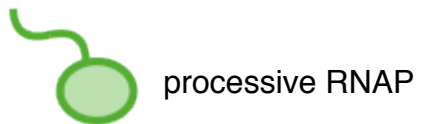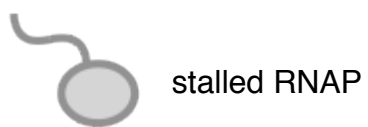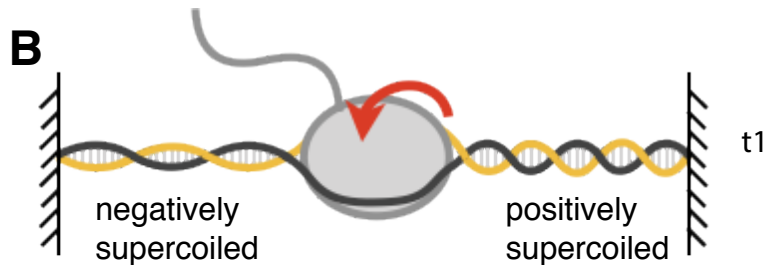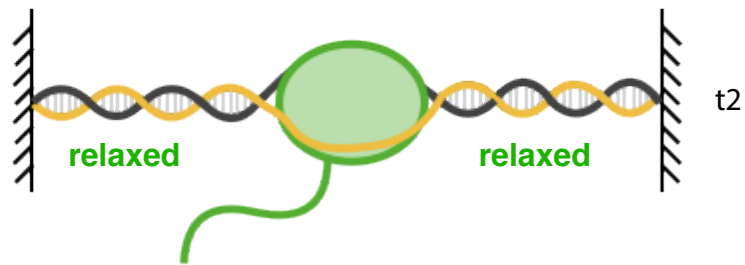

Supplement: S2 Fig — (A) The displacement of RNAP generates negative supercoiling in the upstream region and positive supercoiling in the downstream region. The build-up of these supercoiling could stall a transcribing RNAP molecule. (B) The counterrotation of RNAP (and the associated DNA) could return the DNA to a relaxed state without changing the total linking number of the system. The effect is equivalent to the diffusion of one downstream positive twist to the upstream negative twist to annihilate it. (PDF) [file pcbi.1009788.s002.pdf]

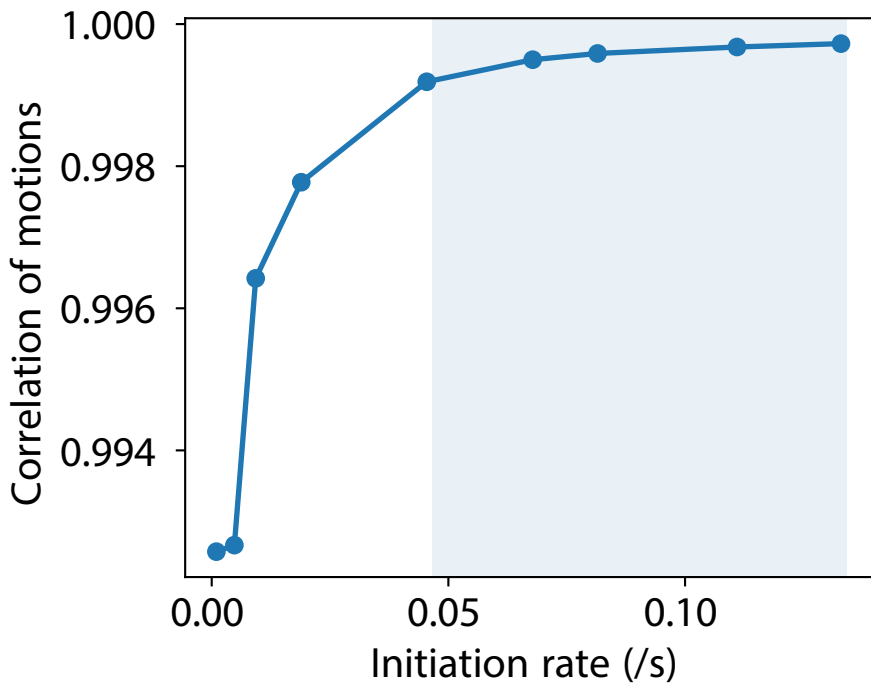

Supplement: S3 Fig — For each condition, 1000 replicates were simulated. Shaded area corresponds to the initiation rates where the apparent elongation rate reaches the maxima. (PDF) [file pcbi.1009788.s003.pdf]

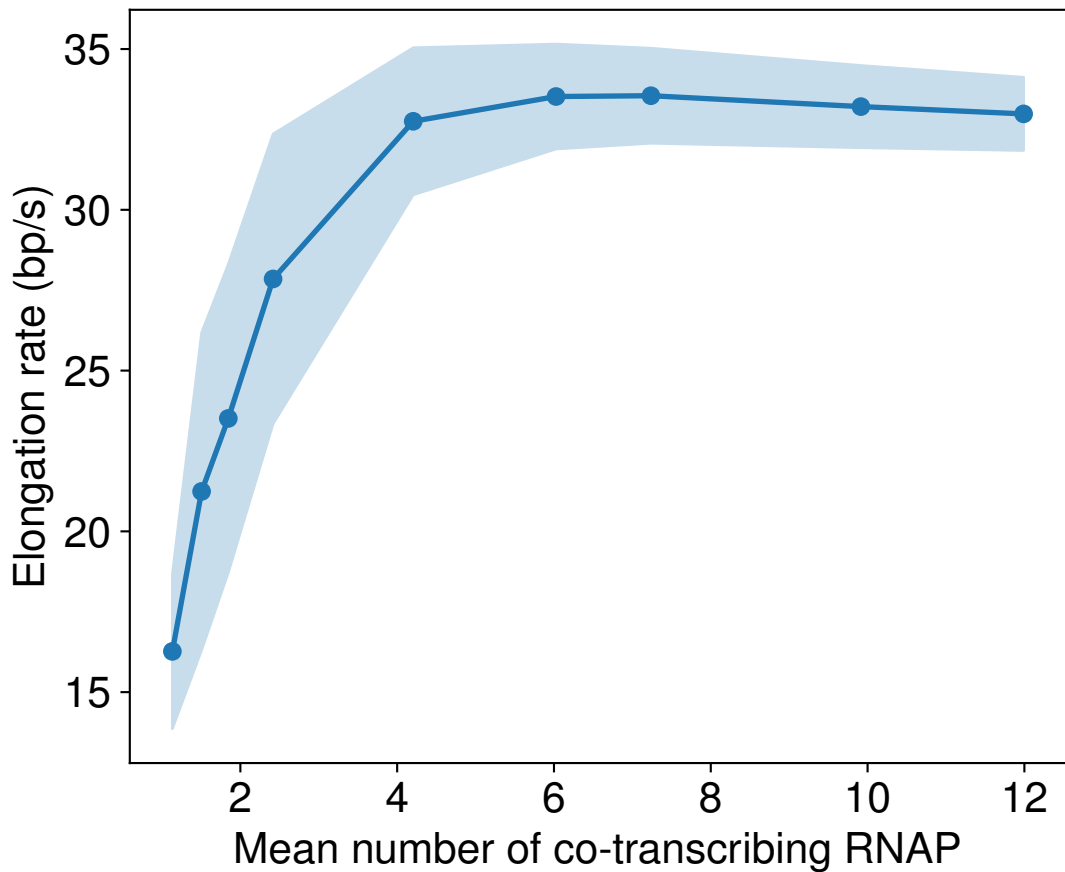

Supplement: S5 Fig — The number of co-transcribing RNAP molecules is only calculated when there is active transcription. The dot is the mean and shaded area is mean ± SD. (PDF) [file pcbi.1009788.s005.pdf]

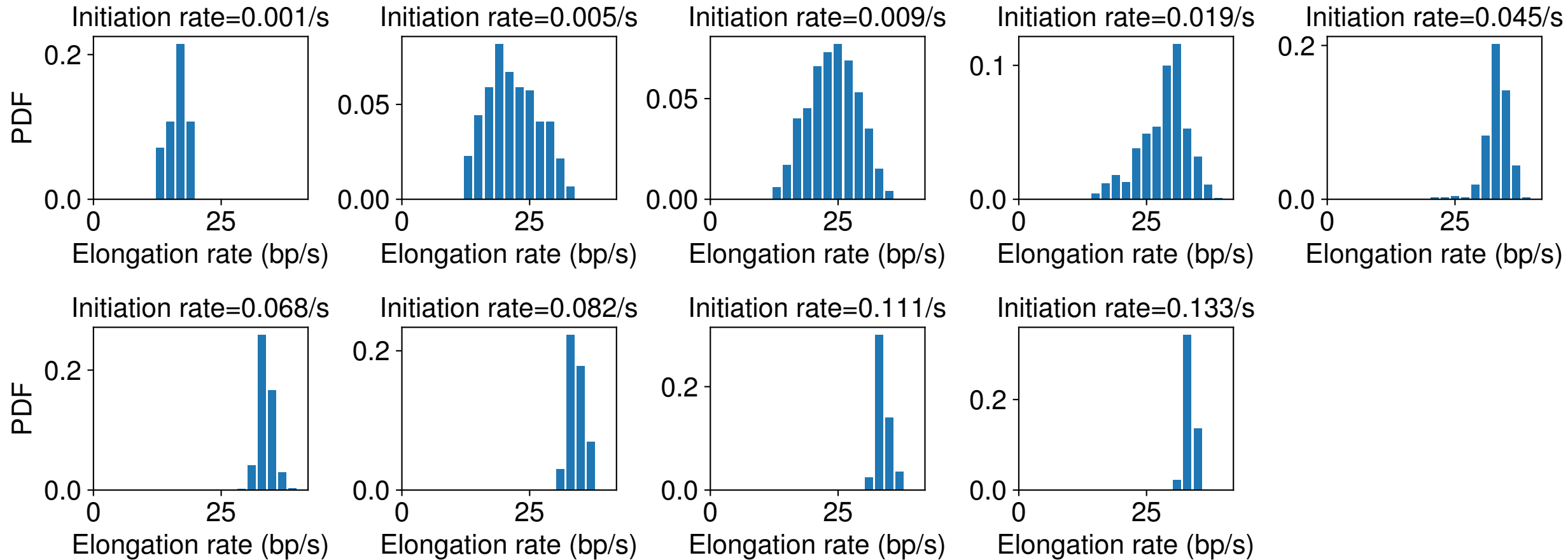

Supplement: S6 Fig — For each condition, 1000 replicates were simulated. The elongation rate is calculated as the transcript length over the duration of the transcription. (PDF) [file pcbi.1009788.s006.pdf]

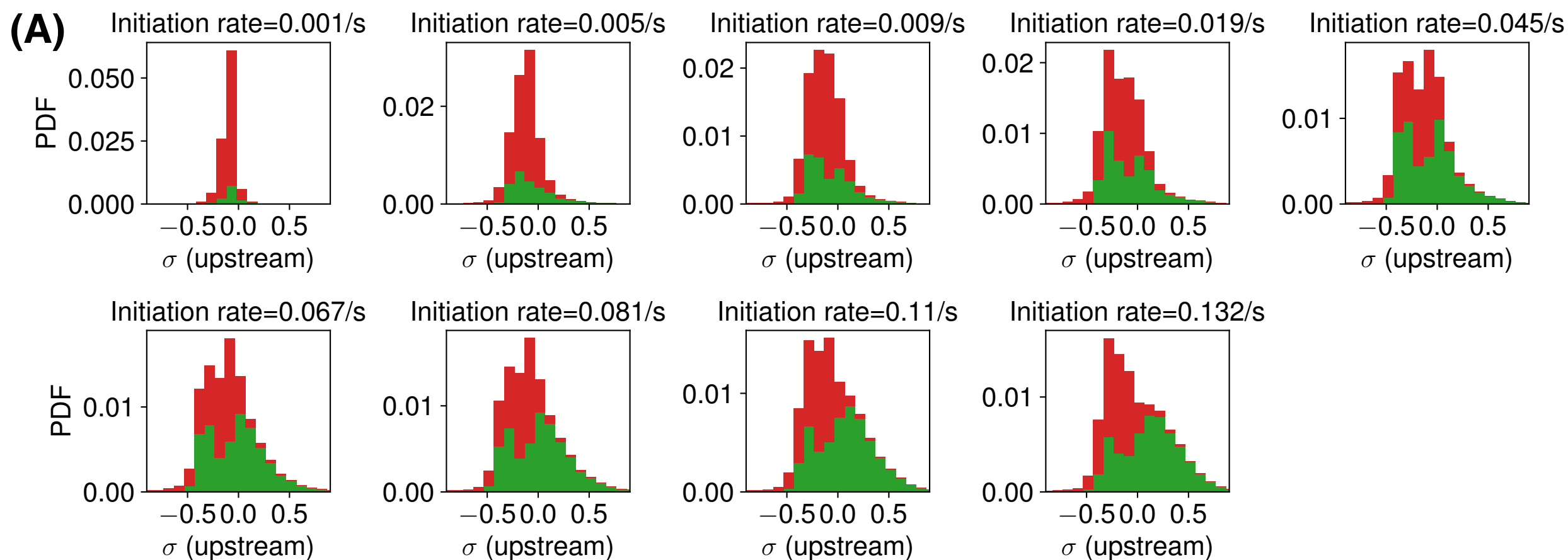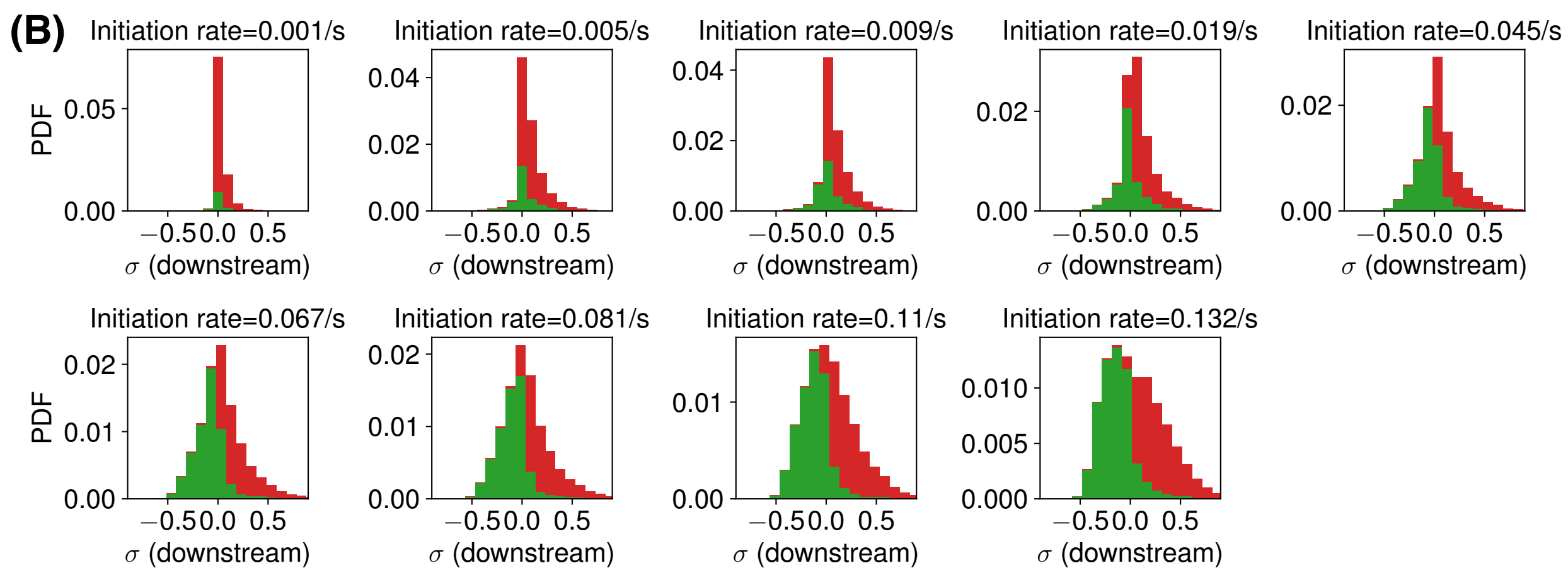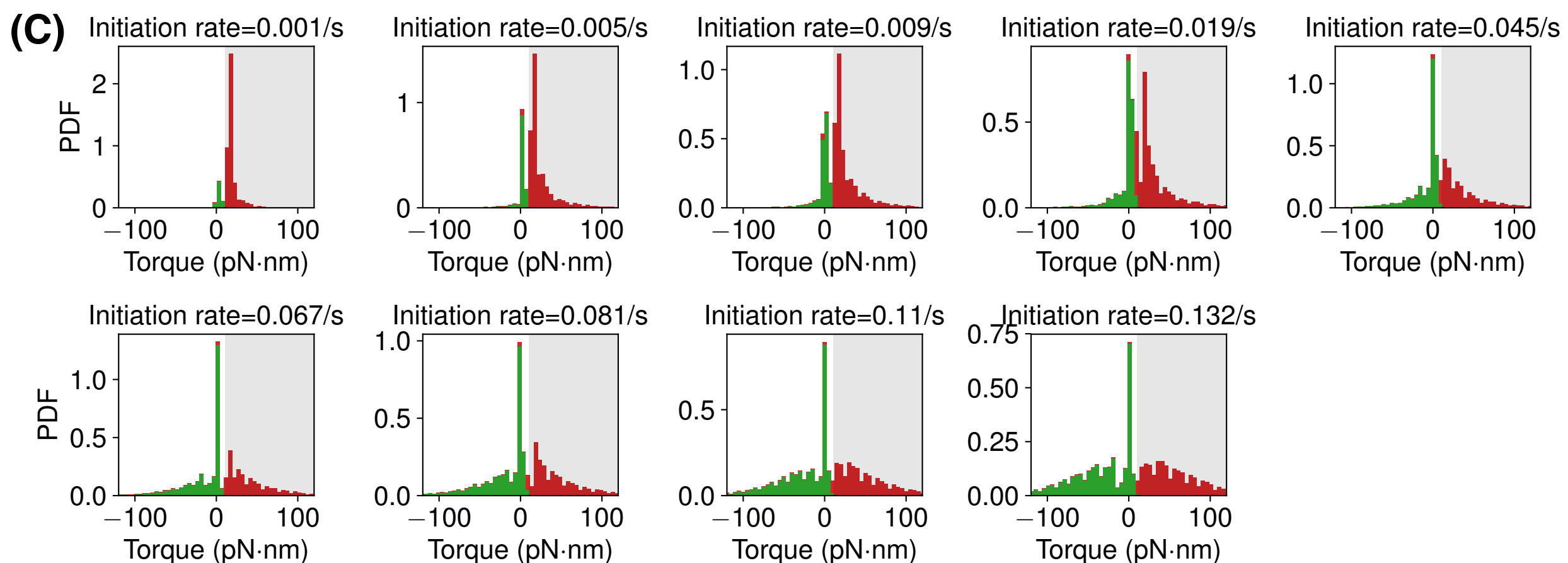

Supplement: S7 Fig — Two populations are shown: processive RNAP (green) and stalled RNAP (red). For each condition, 1000 replicates were simulated. The torque value above the stall threshold is shaded in grey. (PDF) [file pcbi.1009788.s007.pdf]

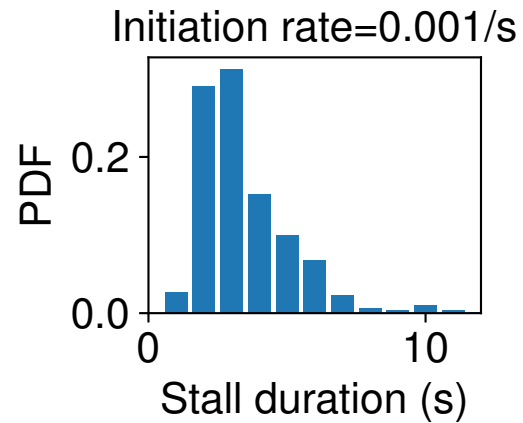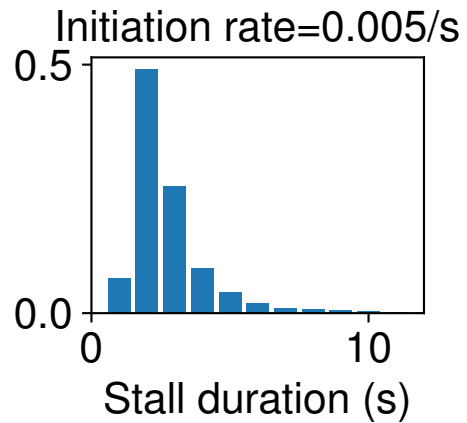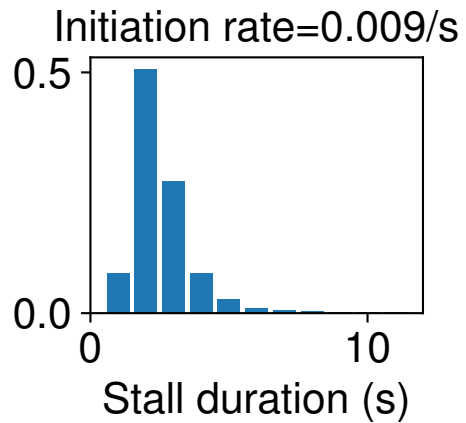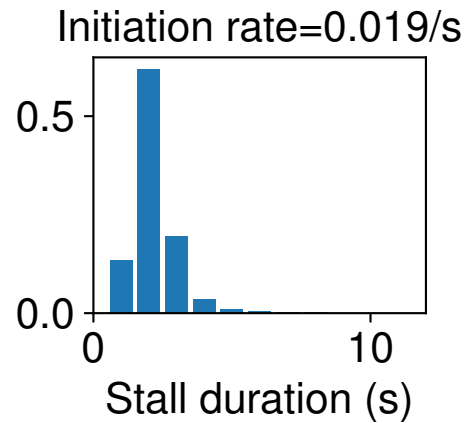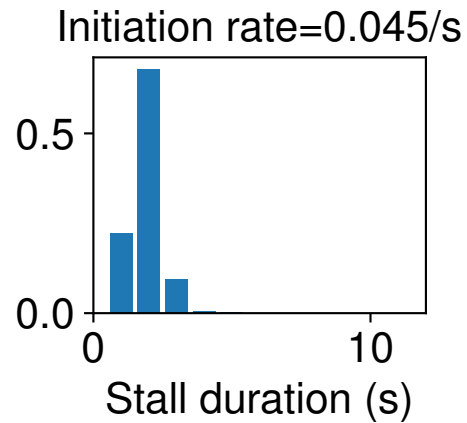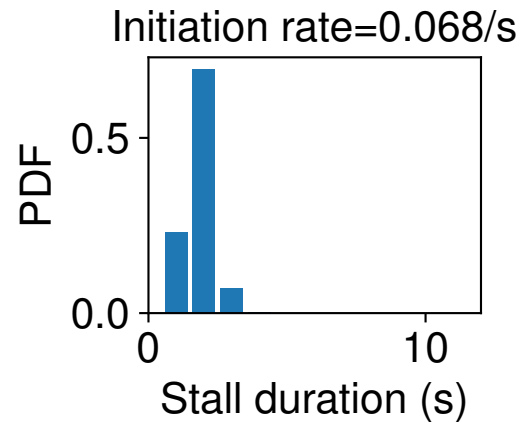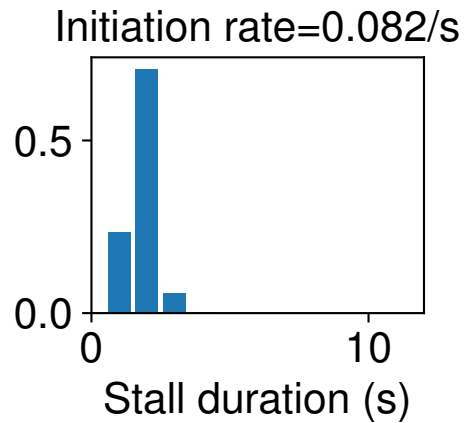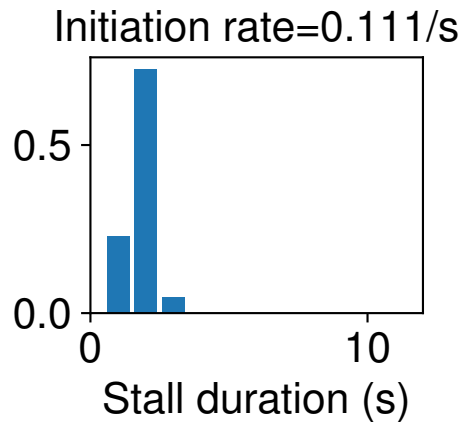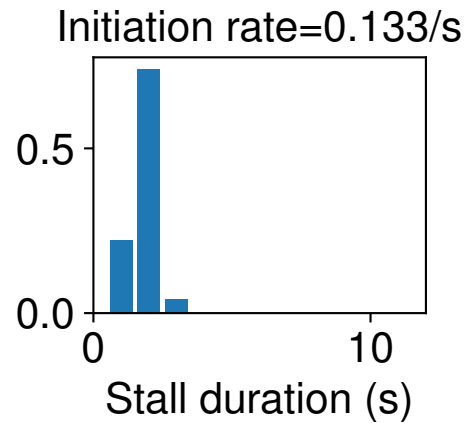

Supplement: S8 Fig — For each condition, 1000 replicates were simulated. (PDF) [file pcbi.1009788.s008.pdf]

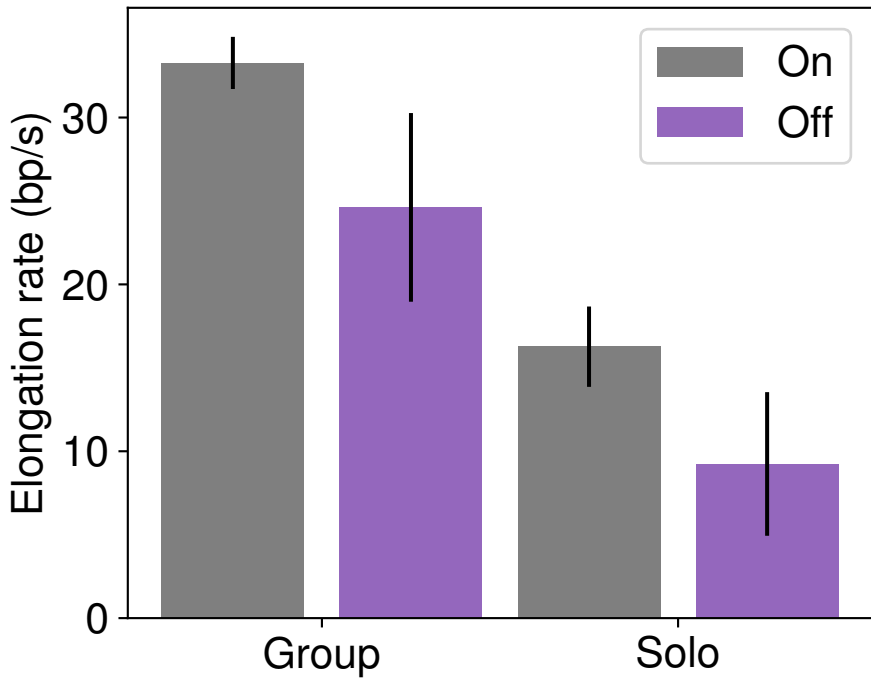

Supplement: S9 Fig — The transcription of a group of RNAP molecules corresponds to a kmax of 0.1 s−1. The transcription of a solo RNAP molecule corresponds to a kmax of 0.001 s−1. Transcription without promoter inactivation is denoted as “On” (grey color). Transcription with promoter inactivation at 2700 bp is denoted as “Off” (purple color). Error bar suggests standard deviation. (PDF) [file pcbi.1009788.s009.pdf]

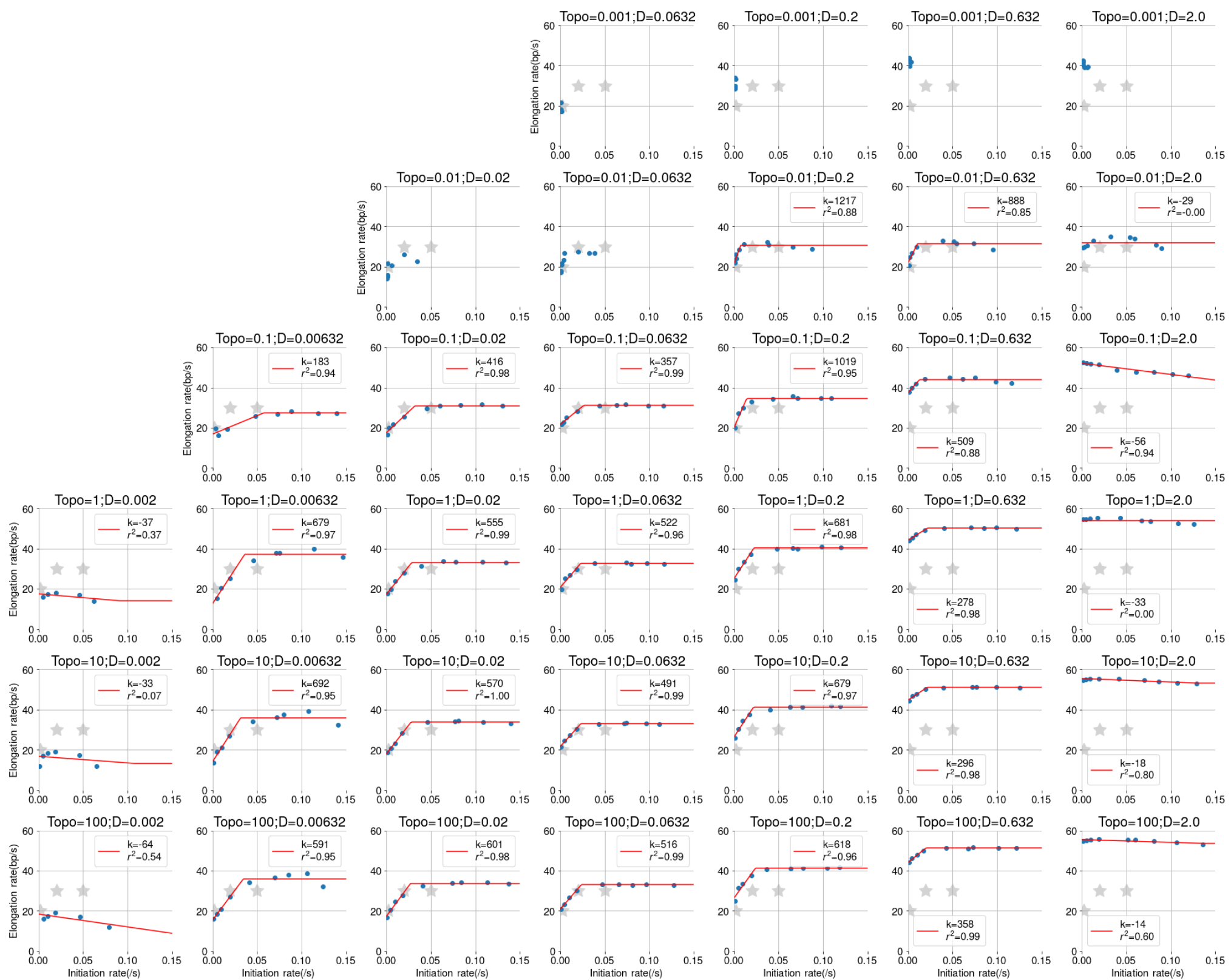

Supplement: S10 Fig — The Topo I unbinding rate is varied from 0.001 to 100 s−1 (row). The supercoiling diffusion rate is varied from 0.002 to 2 μm2 · s−1 (column). For kmax = 0.001 s−1, 500 replicates are simulated. For kmax = 0.005, 0.01, 0.02 s−1, 100 replicated are simulated. For kmax = 0.05, 0.08, 0.1, 0.15, 0.2 s−1, 10 replicates are simulated. The blue dot corresponds to simulated data, and the red curve corresponds to the piece-wise linear fitting. The fitted slope k and R squared value is shown. The grey star corresponds to the experimental data in Kim et al. [1]. (PDF) [file pcbi.1009788.s010.pdf]

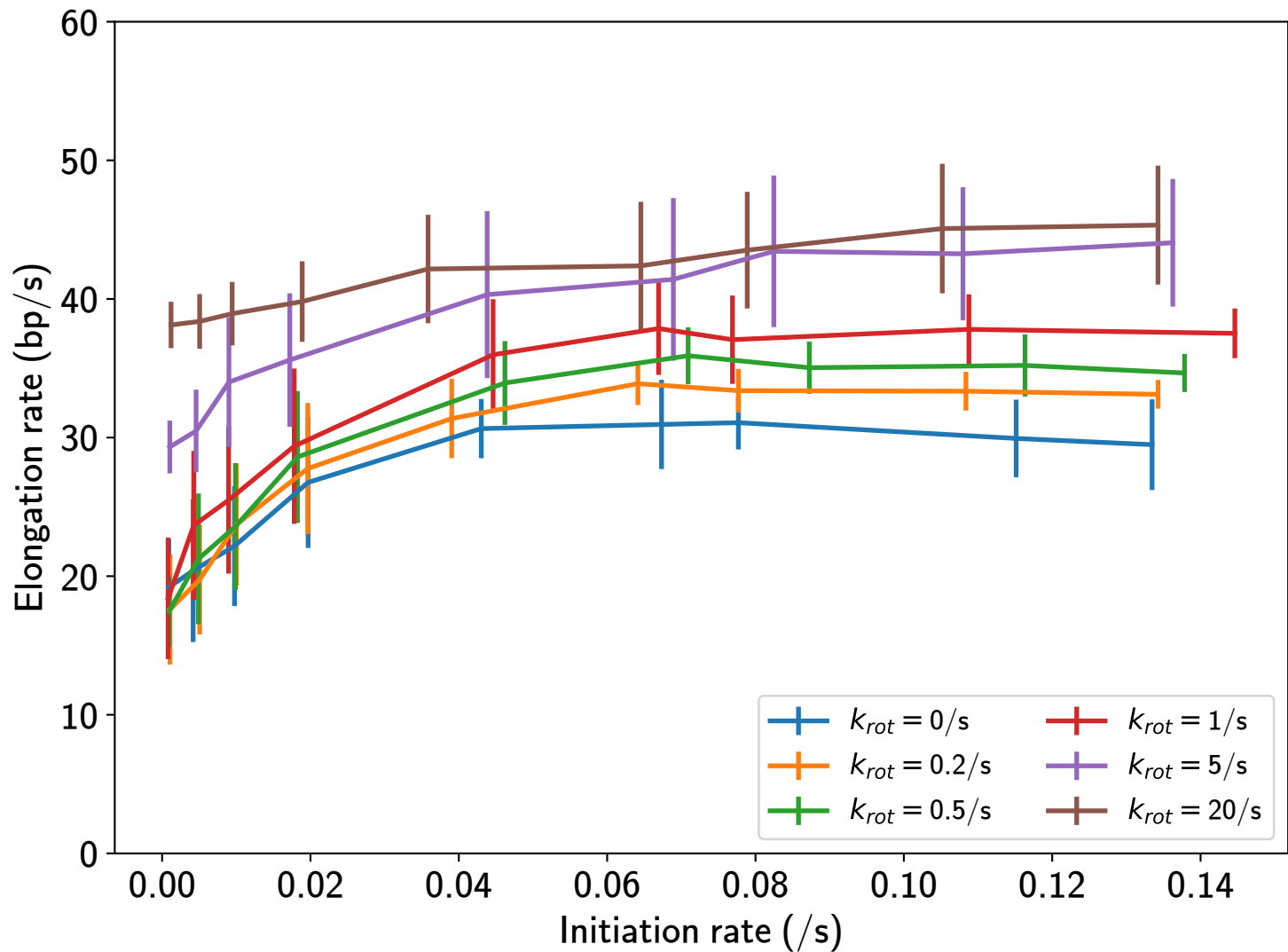

Supplement: S11 Fig — Error bar suggests standard deviation. For kmax = 0.001 s−1, 500 replicates are simulated. For kmax = 0.005, 0.01, 0.02 s−1, 100 replicated are simulated. For kmax = 0.05, 0.08, 0.1, 0.15, 0.2 s−1, 10 replicates are simulated. (PDF) [file pcbi.1009788.s011.pdf]

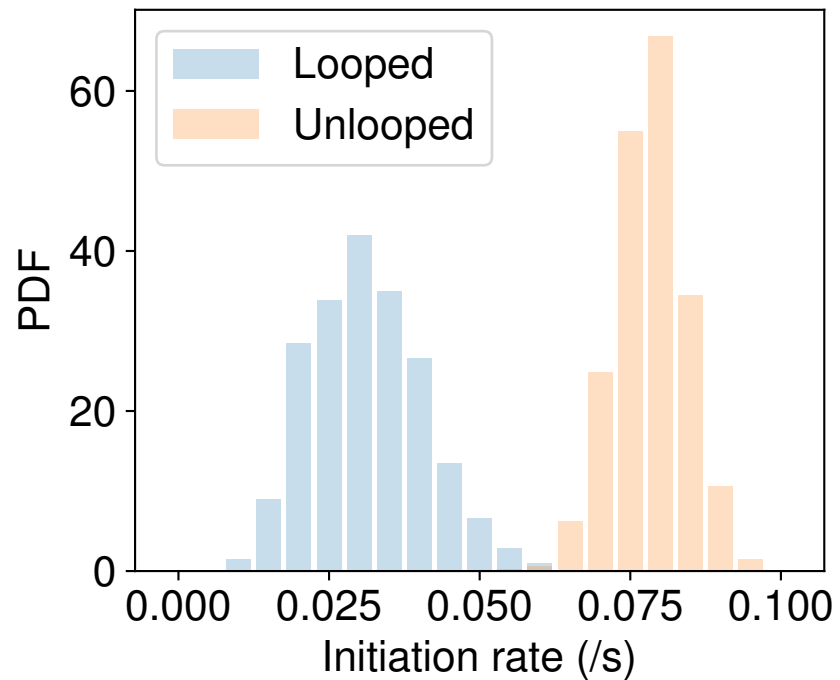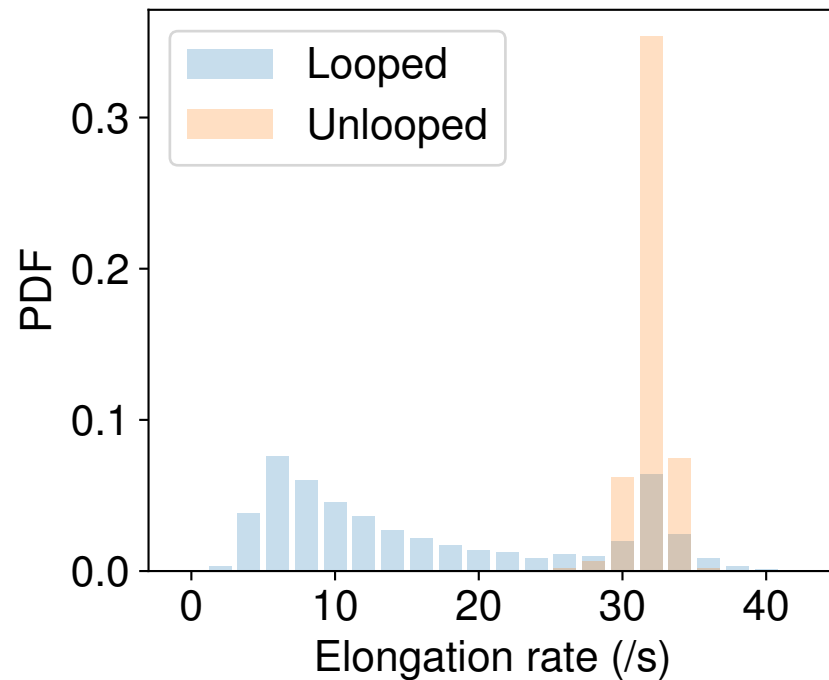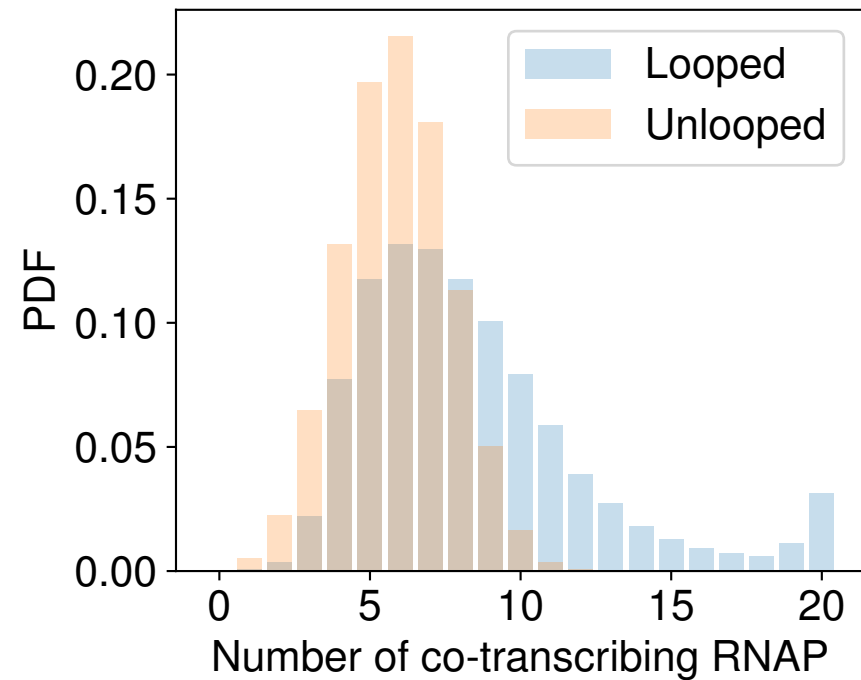

Supplement: S12 Fig — The distribution is drawn from 1000 simulations. Orange bar corresponds to the dynamically looping cased, and blue bar corresponds to the unlooped case. (PDF) [file pcbi.1009788.s012.pdf]

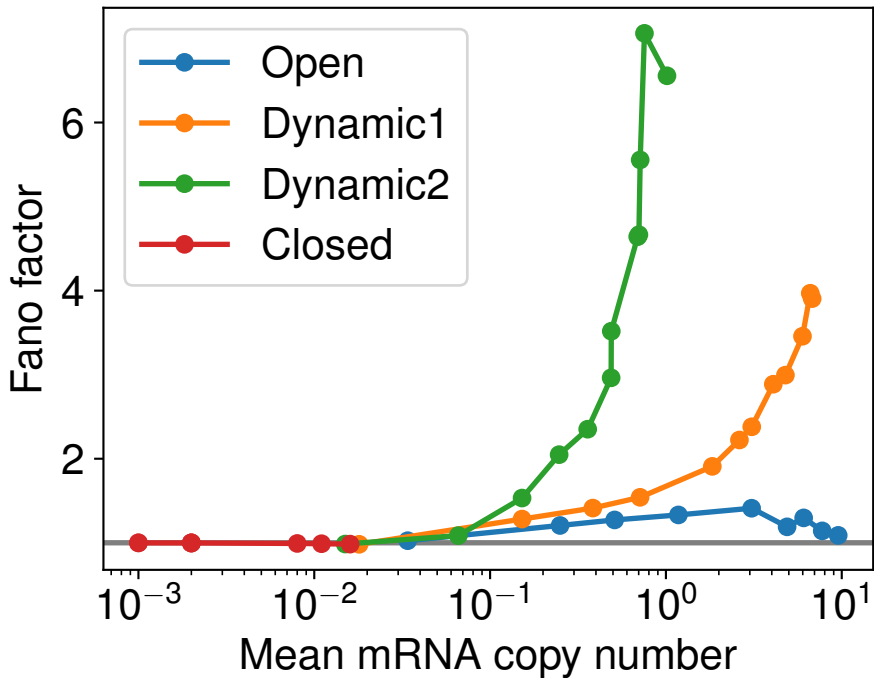

Supplement: S13 Fig — Open (blue): open chromosome end (relaxing to equilibrium every 0.2 s). Dynamic1 (orange): dynamic looped (on average looping for 1 min and unlooping for 1 min). Dynamic2 (green): (on average looping for 5 min and unlooping for 1 min). Closed (red): permanent loop. 1000 replicates are simulated. (PDF) [file pcbi.1009788.s013.pdf]

Convergent

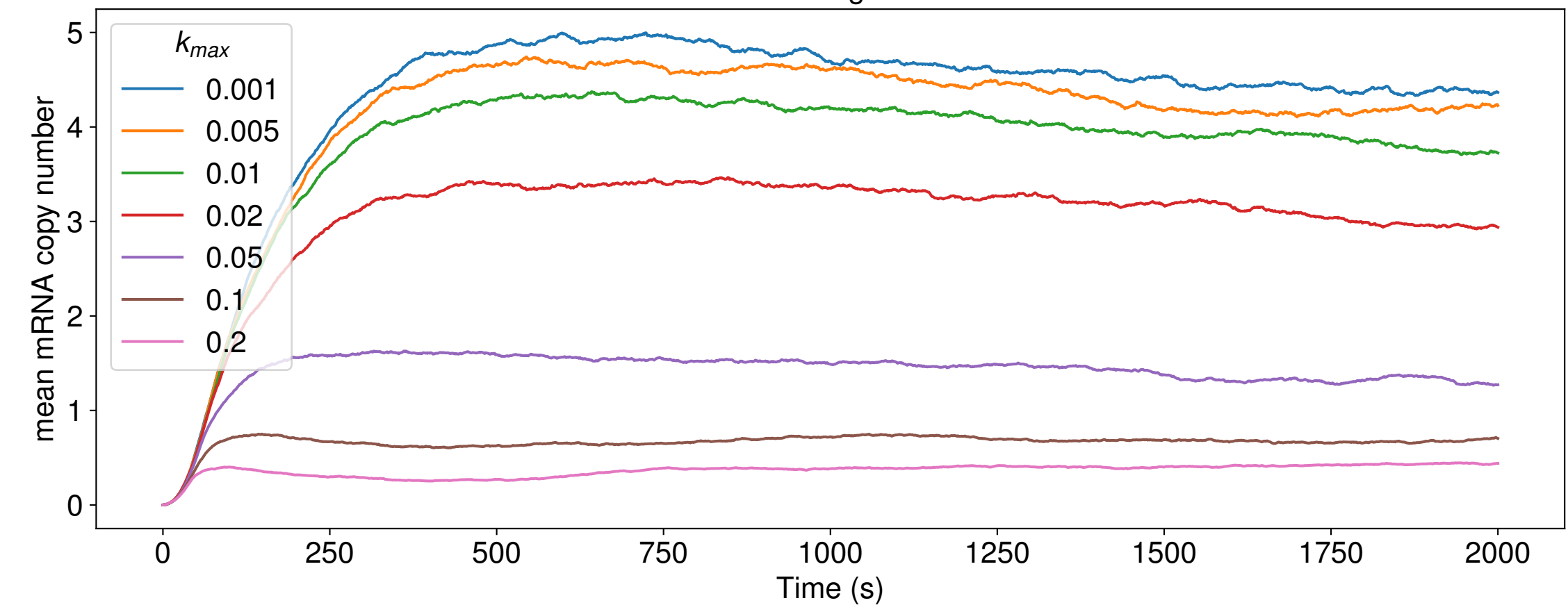

Divergent

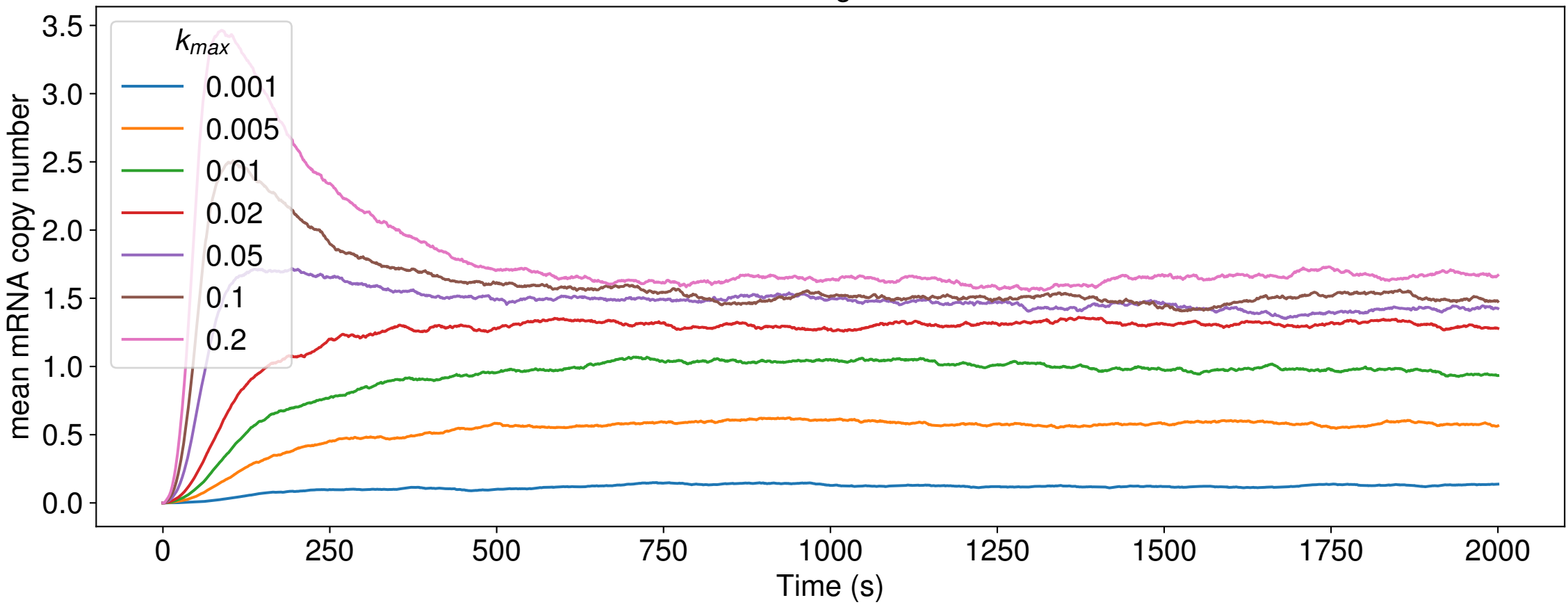

Codirectional-1

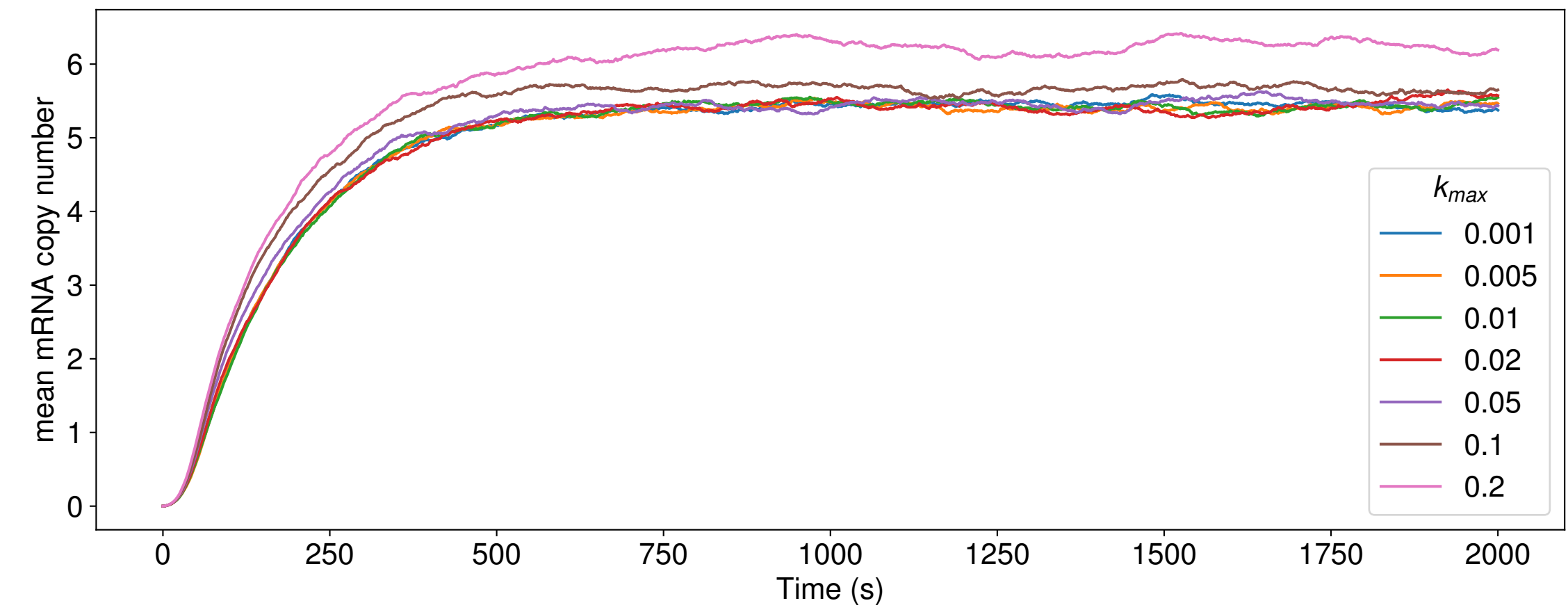

Codirectional-2

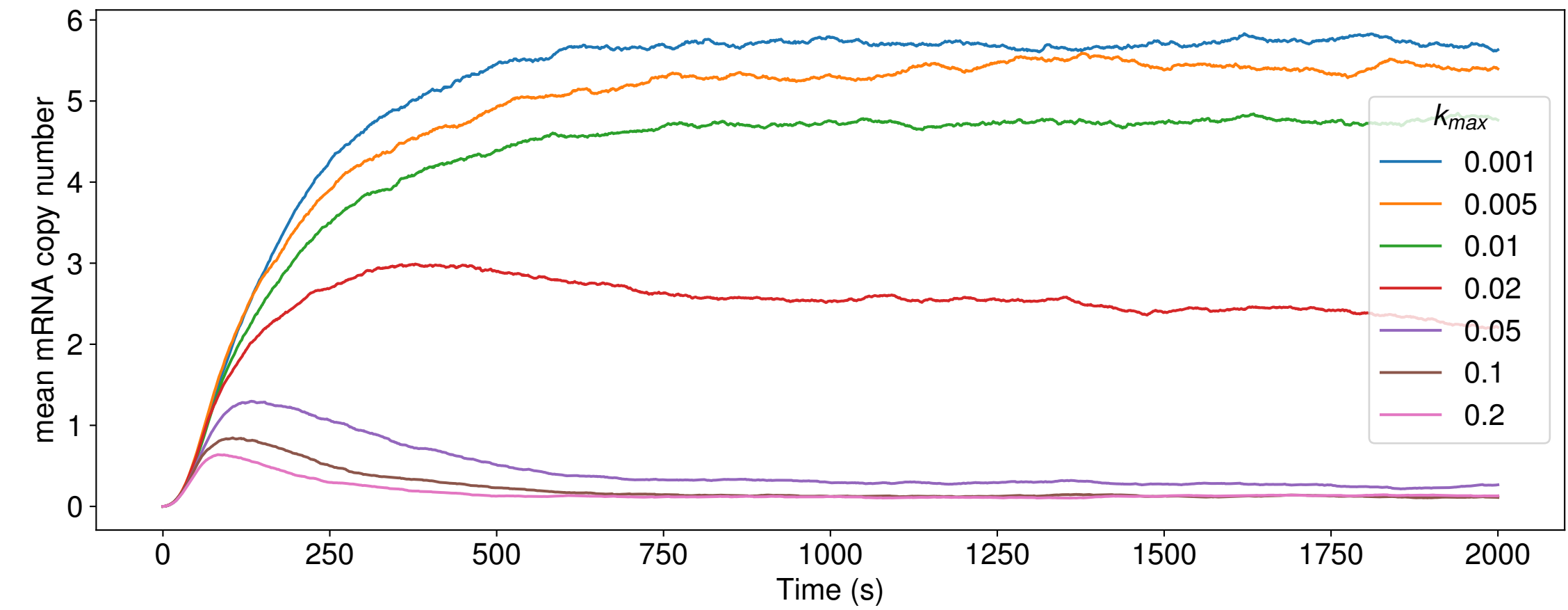

Supplement: S14 Fig — The curve is averaged from 1000 simulations. (PDF) [file pcbi.1009788.s014.pdf]

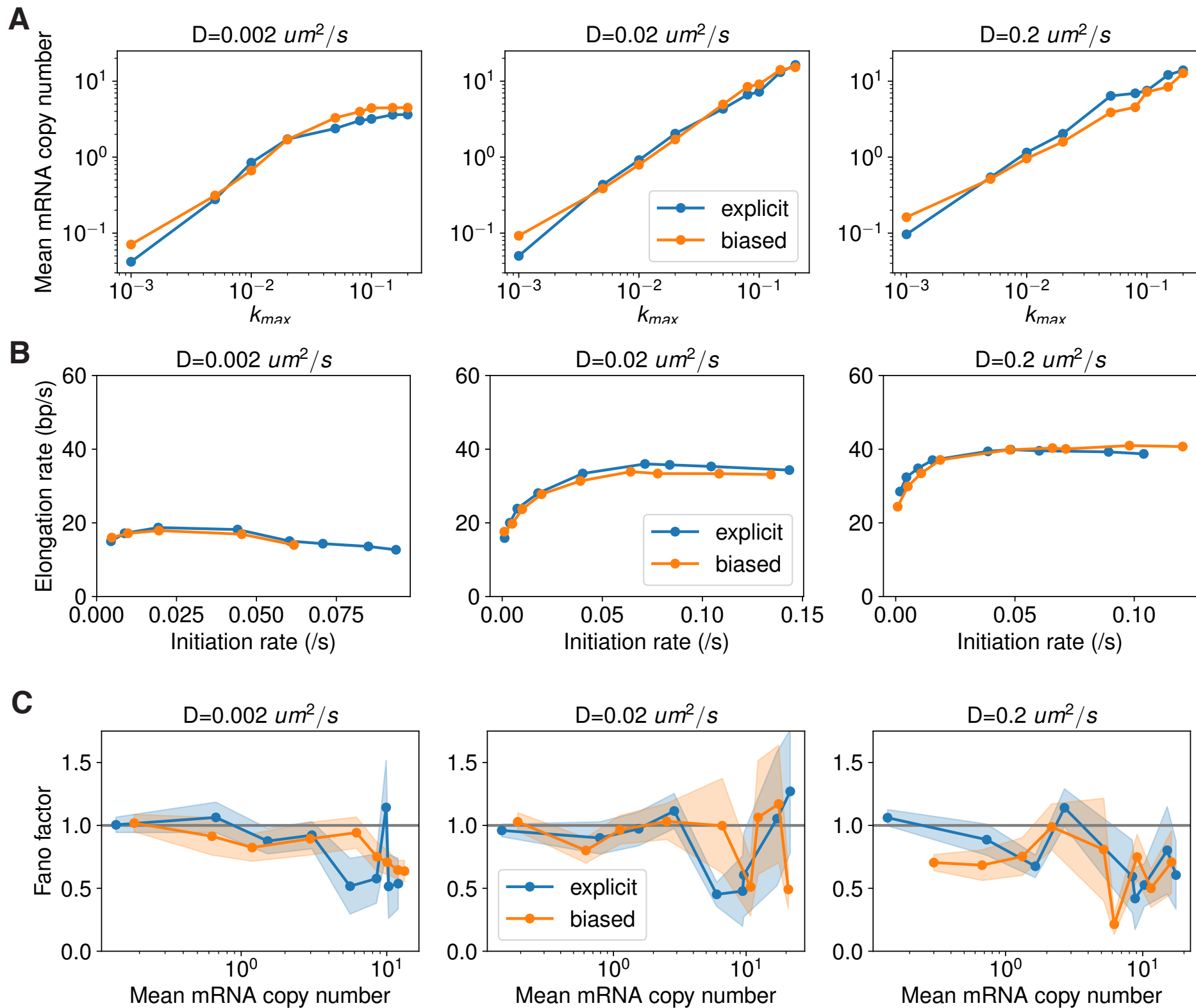

Supplement: S15 Fig — (A) Mean mRNA production as a function of kmax for the biased random walk model (orange) and the explicit random walk model (blue). For kmax = 0.001 s−1, 500 replicates are simulated. For kmax = 0.005, 0.01, 0.02 s−1, 100 replicated are simulated. For kmax = 0.05, 0.08, 0.1, 0.15, 0.2 s−1, 100 replicated are simulated for D = 0.002 μm2 · s−1, and 10 replicates are simulated for D = 0.02 and 0.2 μm2 · s−1. (B) Empirical elongation rate as a function of empirical initiation rate.. (C) Fano factor as a function of mean mRNA copy number. The dot is the mean, and the shaded area is mean ± SEM. (PDF) [file pcbi.1009788.s015.pdf]
