# Supplementary material for: A spatially resolved stochastic model reveals the role of supercoiling in transcription regulation
Source: PLoS Comput Biol. 2022 Sep 19;18(9):e1009788. doi: 10.1371/journal.pcbi.1009788 (PMC9522292; doi:10.1371/journal.pcbi.1009788)
Supplement: S4 Fig — For each condition, 1000 replicates were simulated. From the figures, we could roughly tell that the mean mRNA copy number stabilizes after about 750 s, suggesting that the system reaches a steady state. (PDF) [file pcbi.1009788.s004.pdf]

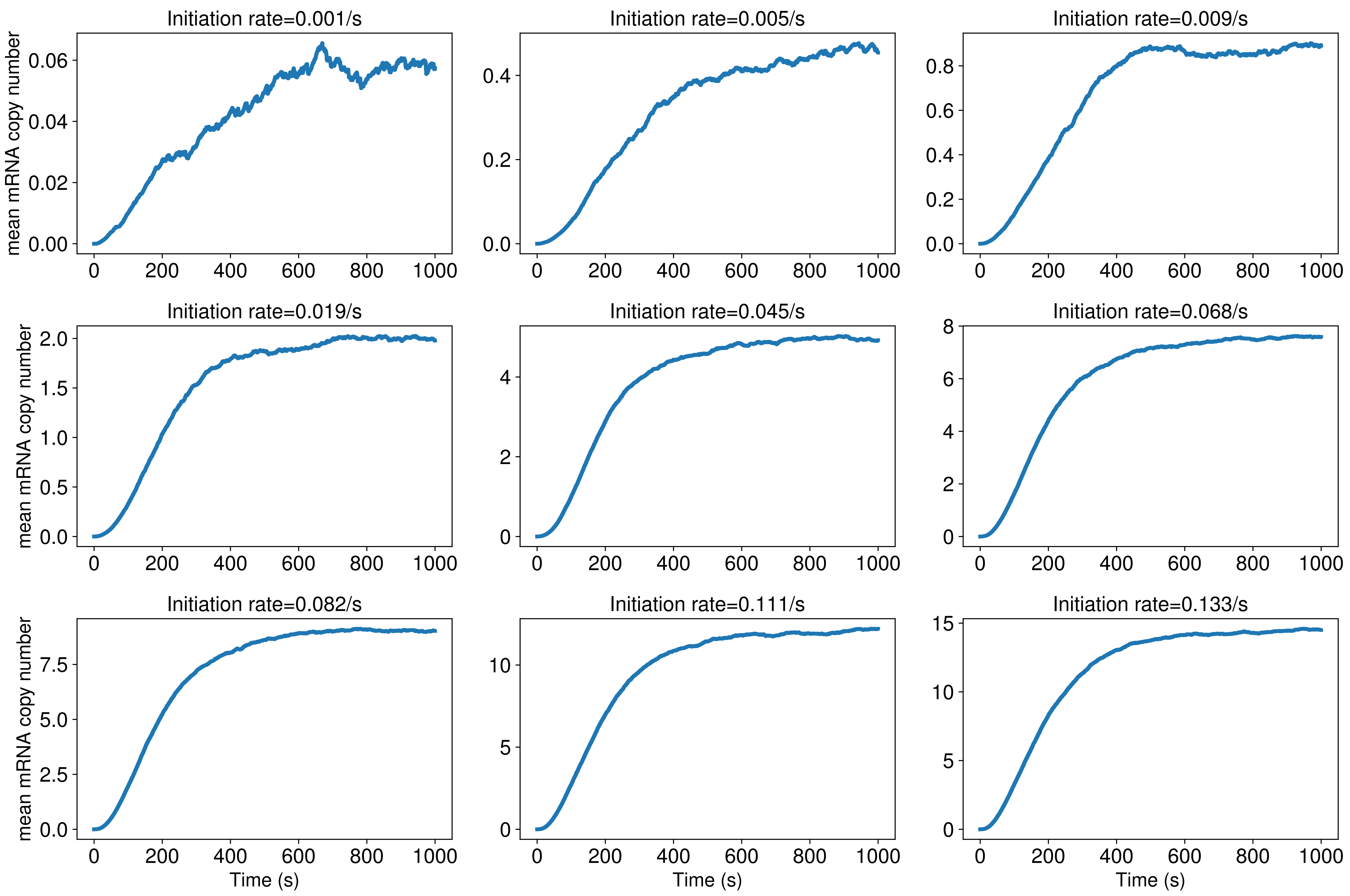

Initiation rate=0.001/s
Initiation rate=0.005/s
Initiation rate=0.009/s
Initiation rate=0.019/s
Initiation rate=0.045/s
Initiation rate=0.068/s
Initiation rate=0.082/s
Initiation rate=0.111/s
Initiation rate=0.133/s
mean mRNA copy number
Time (s)
0
200
400
600
800
1000
