# Supplementary material for: A spatially resolved stochastic model reveals the role of supercoiling in transcription regulation
Source: PLoS Comput Biol. 2022 Sep 19;18(9):e1009788. doi: 10.1371/journal.pcbi.1009788 (PMC9522292; doi:10.1371/journal.pcbi.1009788)
Supplement: S1 Table — (DOCX) [file pcbi.1009788.s016.docx]

**S1 Table. Species index**

| **Index** | **Species** |
| --- | --- |
| 0-(num-1) | DNA |
| num-(2num-1) | RNAP |
| 2num-(3num-1) | RNAP_stall |
| 3num-(4num-1) | Turn |
| 4num-(5num-1) | Gyrase_unbind |
| 5num-(6num-1) | Gyrase_bind |
| 6num-(7num-1) | TopoI-unbind |
| 7num-(8num-1) | TopoI_bind |
| 8num-(9num-1) | RNAP_tmp |
| 9num-(10num-1) | step |
| 10num-(11num-1) | mRNA |
| 11num-(12num-1) | mRNA_degr |
| (12num)-(12num+m-1) | S1 |
| (12num+m)-(12num+2m-1) | S2 |
| 12num+2m | loop_state |
| 12num+2m+1 | unloop_state |
| 12num+2m+1 | loop1 |
| 12num+2m+3 | loop2 |
| 12num+2m+4 | unloop1 |
| 12num+2m+5 | unloop2 |
