# Supplementary material for: A spatially resolved stochastic model reveals the role of supercoiling in transcription regulation
Source: PLoS Comput Biol. 2022 Sep 19;18(9):e1009788. doi: 10.1371/journal.pcbi.1009788 (PMC9522292; doi:10.1371/journal.pcbi.1009788)
Supplement: S2 Table — (DOCX) [file pcbi.1009788.s017.docx]

**S2 Table. Model equations**

| Description | Reactions | Propensity |
| --- | --- | --- |

**Transcription initiation, elongation and termination**

| Transcription initiation | | DNA(P) → RNA(P+1)+S1 *f_a_*(Turn(P)) · DNA(P) · DNA(P+1) | |  |  |
| --- | --- | --- | --- | --- | --- |
| RNAP elongation | | RNAP(k) → RNAP(k)+step(k) *k_elongation_* · RNAP(k) | |  |  |
| RNAP stall | | RNAP(k) → RNAP_stall(k) *f_s_*(Turn(k-1),Turn(k+1)) · RNAP(k) | |  |  |
| Stall resumption | | RNAP_stall(k) → RNAP(k) *f_r_*(Turn(k-1),Turn(k+1)) · RNAP_stall(k) | |  |  |
| Translocation-1 | | RNAP(k)+60step(k) → RNAP_tmp(k) *Inf* · RNAP(k) · step(k) | |  |  |
| Translocation-2 | | RNAP_tmp(k)+DNA(k+1) → RNAP(k+1)+DNA(k)+mRNA(k) *Inf* · RNAP_tmp(k) · DNA(k+1) · DNA(k+2) | |  |  |
| Translocation-3 | | Turn(k) → Turn(k+1) *Inf* · Turn(k) · RNAP(k) | |  |  |
| RNAP rotation | | Turn(k+1) → Turn(k-1) *k_rot_* · RNAP*(k) · Turn(k+1) | |  |  |
| RNAP rotation | | Turn(k-1) → Turn(k+1) *k_rot_* · RNAP*(k) · Turn(k-1) | |  |  |
| Termination | | RNAP(T) → DNA(T)+mRNA(T)+S2 *Inf* · RNAP(T)    **mRNA degradation** | |  |  |
| mRNA degradation-1 | | mRNA(P) → mRNA_degr(P+1) *k_mdegr_* · mRNA(P) | |  |  |
| mRNA degradation-2 | | mRNA(k)+mRNA_degr(k) → mRNA_degr(k+1) *Inf* · mRNA(k) · mRNA_degr(k) | |  |  |
| mRNA degradation-3 | | mRNA(T)+mRNA_degr(T) → ∅ *Inf* · mRNA(T) · mRNA_degr(T) | |  |  |
| mRNA degradation-4 | | mRNA_pre(k)+mRNA_degr(k) → ∅ *Inf* · mRNA pre(k) · mRNA_degr(k) | |  |  |
|  | | **Supercoiling diffusion, birth and death at chromosome end** | |  |  |
| Turn diffusion-1 | | Turn(k) → Turn(k+1) *k_drift_* · *f_d_*(Turn(k)-Turn(k+1)) · DNA(k+1) | |  |  |
| Turn diffusion-2 | | Turn(k) → Turn(k-1) *k_drift_* · *f_d_*(Turn(k)-Turn(k-1)) · DNA(k-1) | |  |  |
| Turn birth | | ∅ → Turn(end) *k_birth_* · DNA(end) | |  |  |
| Turn death | | Turn(end) → ∅ *k_death_* · Turn(end)  **Topoisomerase binding, catalysis and dissociation** | |  |  |
| Gyrase binding | | Gyrase_unbind(k)+DNA(k) → Gyrase bind(k) *k_gbind_* · Gyrase_unbind(k) · DNA(k) | |  |  |
| Gyrase catalysis | | Turn(k) → ∅ *k_gcat_* · *f_g_*(Turn(k)) · Gyrase_bind(k) | |  |  |
| Gyrase dissociation | | Gyrase_bind(k) → Gyrase_unbind(k)+DNA(k) *k_gdis_* · Gyrase_bind(k) | |  |  |
| Topo I binding | | TopoI_unbind(k)+DNA(k) → TopoI_bind(k) *k_tbind_* · TopoI_unbind(k) · DNA(k) | |  |  |
| Topo I catalysis | | ∅ → Turn(k) *k_tcat_* · *f_g_*(Turn(k)) · TopoI_bind(k) | |  |  |
| Topo I dissociation | | TopoI_bind(k) → TopoI_unbind(k)+DNA(k) *k_tdis_* · TopoI_bind(k)  **Topological domain formation and dissociation** | |  |  |
| loop formation-1 | | unloop_state → loop1+loop2+loop_state *k_loop_* · unloop_state | |  |  |
| loop formation-2 | | loop1+DNA(site1) → ∅ *Inf* · loop1 · DNA(site1) | |  |  |
| loop formation-3 | loop2+DNA(site2) → ∅ | *Inf* · loop2 · DNA(site2) | |  |  |
| loop dissociation-1 | loop_state → unloop1+unloop2+unloop_state | *k_unloop_* · unloop_state | |  |  |
| loop dissociation-2 | unloop1 → DNA(site1) | *Inf* · unloop1 | |  |  |
| loop dissociation-3 | | | unloop2 → DNA(site2) | *Inf* · unloop2 | |

Note that RNAP* indicate all kinds of RNAP species (i.e., RNAP, RNAP_tmp, RNA_stall)

P stands for the index of promoter site. T stands for the index of terminator site.

S1 is the indicator for transcription initiation, and S2 is the indicator for transcription termination.
