# Supplementary material for: A spatially resolved stochastic model reveals the role of supercoiling in transcription regulation
Source: PLoS Comput Biol. 2022 Sep 19;18(9):e1009788. doi: 10.1371/journal.pcbi.1009788 (PMC9522292; doi:10.1371/journal.pcbi.1009788)
Supplement: S3 Table — (DOCX) [file pcbi.1009788.s018.docx]

**S3 Table. Parameter estimation**

| Notation | Description | Value | Reference |
| --- | --- | --- | --- |
| *kelongation* | RNAP elongation rate rotation | 60 bp·*s*^−1^ | [1] |
| *krot* | rate of RNAP rotation | 0.2 *s*^−1^ | free parameter |
| *kmdegr* | mRNA degradation rate | 0.0067 *s*^−1^ | [1] |
| *kdrift* | supercoiling drift rate | 50 *s*^−1^ | [2] |
| *kbirth* | supercoiling birth rate at chromosome end | 50 *s*^−1^ | free parameter |
| *kdeath* | supercoiling death rate at chromosome end | 1 *s*^−1^ | free parameter |
| *kgbind* | Gyrase binding rate | 0.0018 *s*^−1^ | [3] |
| *kgcat* | Gyrase catalytic rate | 8.4 *s*^−1^ | [3] |
| *kgdis* | Gyrase dissociation rate | 0.4 *s*^−1^ | [3] |
| *ktbind* | Topo I binding rate | 0.0018 *s*^−1^ | [3, 4] |
| *ktcat* | Topo I catalytic rate | 4.2 *s*^−1^ | [3] |
| *ktdis* | Topo I dissociation rate | 1 *s*^−1^ | free parameter |
| *kloop* | loop formation rate | 0.00833 *s*^−1^ | free parameter |
| *kunloop* | loop dissociation rate | 0.00167 *s*^−1^ | free parameter |

## References

1. Chen H, Shiroguchi K, Ge H, Xie XS. Genome‐wide study of mRNA degradation and transcript elongation in E scherichia coli. Molecular systems biology. 2015 Jan;11(1):781.
2. van Loenhout MT, De Grunt MV, Dekker C. Dynamics of DNA supercoils. Science. 2012 Oct 5;338(6103):94-7.
3. Stracy M, Wollman AJ, Kaja E, Gapinski J, Lee JE, Leek VA, McKie SJ, Mitchenall LA, Maxwell A, Sherratt DJ, Leake MC. Single-molecule imaging of DNA gyrase activity in living Escherichia coli. Nucleic acids research. 2019 Jan 10;47(1):210-20.
4. Taniguchi Y, Choi PJ, Li GW, Chen H, Babu M, Hearn J, Emili A, Xie XS. Quantifying E. coli proteome and transcriptome with single-molecule sensitivity in single cells. science. 2010 Jul 30;329(5991):533-8.
