# Supplementary material for: A spatially resolved stochastic model reveals the role of supercoiling in transcription regulation
Source: PLoS Comput Biol. 2022 Sep 19;18(9):e1009788. doi: 10.1371/journal.pcbi.1009788 (PMC9522292; doi:10.1371/journal.pcbi.1009788)
Supplement: S4 Table — (DOCX) [file pcbi.1009788.s019.docx]

**S4 Table. Empirical initiation rate, elongation rate and RNAP density under different promoter strengths**

| *k_max_* (/sec) | 0.001 | 0.005 | 0.01 | 0.02 | 0.05 | 0.08 | 0.1 | 0.15 | 0.2 |
| --- | --- | --- | --- | --- | --- | --- | --- | --- | --- |
| Mean empirical initiation rate (/sec) | 0.001 | 0.005 | 0.009 | 0.019 | 0.045 | 0.068 | 0.082 | 0.111 | 0.133 |
| Empirical elongation rate (bp/s) (mean ± SD) | 16.3 ± 2.4 | 21.2 ± 4.9 | 23.5 ± 4.8 | 27.9 ± 4.5 | 32.8 ± 2.3 | 33.5 ±1.6 | 33.5 ±1.5 | 33.2 ±1.3 | 33.0 ±1.1 |
| Mean RNAP number on *lacZ* gene | 1.1 ± 0.4 | 1.5 ± 0.7 | 1.8 ± 0.9 | 2.4 ± 1.2 | 4.2 ± 1.8 | 6.0 ± 2.1 | 7.2 ± 2.2 | 9.9 ± 2.2 | 12.0 ± 2.1 |
