# Supplementary material for: A spatially resolved stochastic model reveals the role of supercoiling in transcription regulation
Source: PLoS Comput Biol. 2022 Sep 19;18(9):e1009788. doi: 10.1371/journal.pcbi.1009788 (PMC9522292; doi:10.1371/journal.pcbi.1009788)
Supplement: S1 File — (DOCX) [file pcbi.1009788.s020.docx]

# S1 File. Supplementary Notes

## 1. Basic modeling strategy

We adopted a stochastic framework since transcription happens in the low copy number regime. Chemical master equations are typically used to describe reactions between species in a probabilistic setting. However, traditional chemical master equations apply to well-stirred systems and cannot capture the spatial heterogeneity of molecules. To overcome this, we partitioned the DNA into segments of a fixed size. Each segment has several attributes (for example, the availability of DNA, the occupation of normal RNAP, the occupation of stalled RNAP and the number of turns), and different attributes at each segment is represented by different species. The evolution of attributed at different segments can be represented by different reactions. Attributes can be either binary or integral.

In the case mentioned above, the availability of DNA, the occupation of normal RNAP and the occupation of stalled RNAP are binary attributes, and they are mutually exclusive. If *RNAP*(*k*) = 1, both *DNA*(*k*) and *RNAP stalll*(*k*) will be 0, since the DNA at k-th segment has been occupied by RNA, and the RNAP at k-th segment can only take one state, either normal or stalled. Similarly, if *DNA*(*k*) = 1, both *RNAP*(*k*) and *RNAP stalll*(*k*) will be 0, since *DNA*(*k*) = 1 suggests that no proteins occupy the k-th segment. The number of turns is an integral attributed: if *Turn*(1) = 5, it means that 5 turns wraps around the first DNA segment, and we can calculate the supercoiling density accordingly.


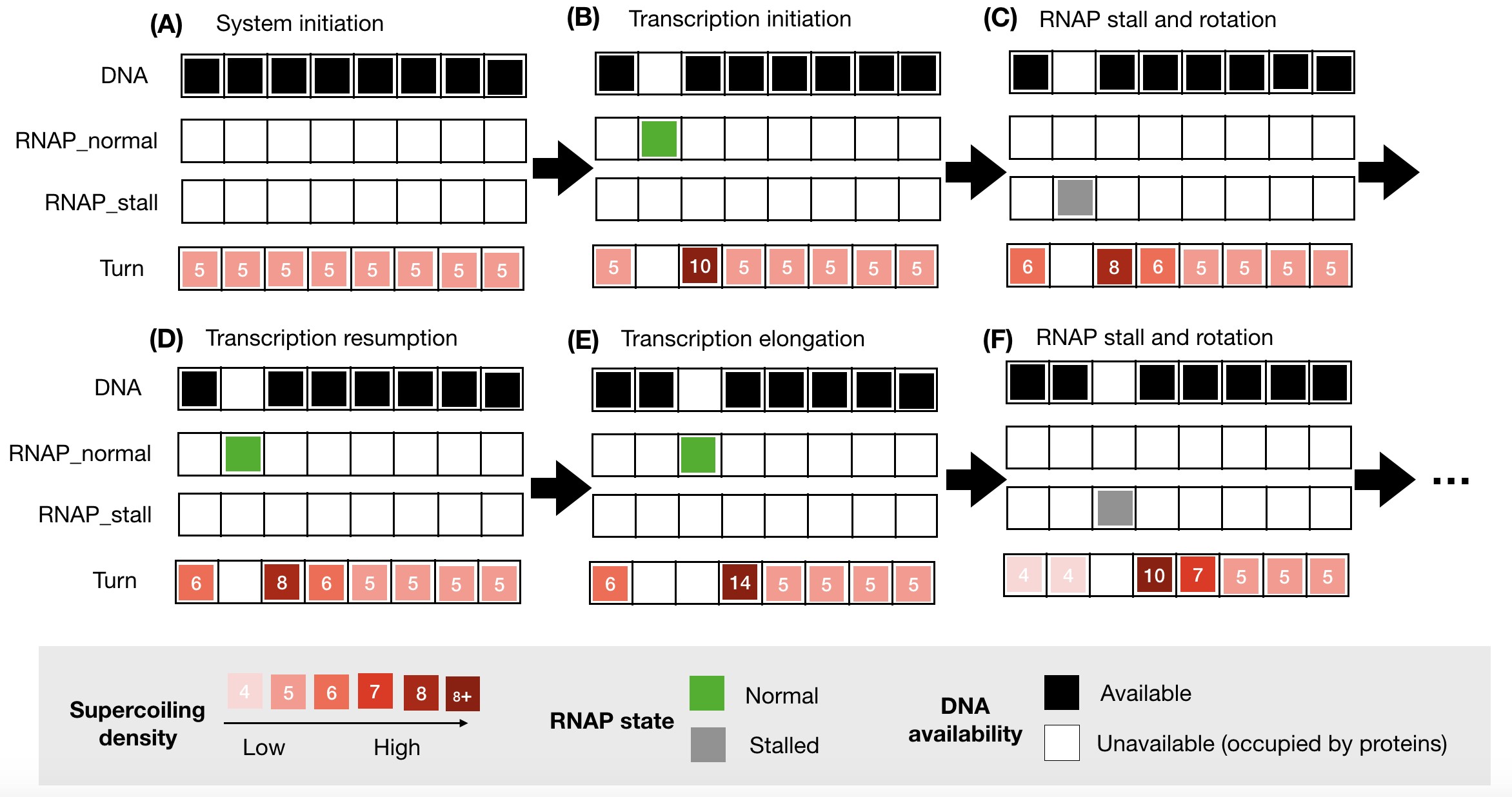


**S16 Fig. A toy model that shows how spatially resolved model works.** (A) The system is initialized with all DNA available and evenly distributed turns. (B) Once transcription initiates, the 2nd DNA segment is occupied and the turns are pushed forward. (C) Due to the high torsional stress, RNAP switches to the stalled state and allows the turns to diffuse over it. (D) Due to the reduced torsional stress, the RNAP switches back to the normal state and is ready for translocation. (E) The RNAP translocates to the 3rd segment and pushes turns forward. (F) The RNAP switches to the stalled state and allows the turns to diffuse over it.

This framework shown above is very flexible and can be applied to any spatially resolved dynamical system. The drawback is that as we increase the number of the segments we simulate, the number of species and reactions grow proportionally, increasing the simulation time. To balance the accuracy and computational load, we partitioned the DNA into 60-bp segments. We choose 60-bp as the unit of positions since it is at the same magnitude of the size of Gyrase binding sites (137 bp) [1] and RNAP footprints (32 bp) [2].

## 2. Characterization of topoisomerase response to supercoiling

Both Gyrase and Topo I are supercoiling-sensitive. It has been shown that Gyrase removes positive supercoils faster than it introduces negative supercoils in relaxed DNA [3]. Moreover, Topo I could only work when DNA is negatively supercoiled [4]. In this work, since we only care about the effects of topoisomerase on the supercoiling density, the detailed kinetics were not taken into consideration (like the multiple modes of Gyrase [5]). Instead, we used phenomenological models to characterize the overall activity of topoisomerase in response to DNA supercoiling density. We assumed that the unbinding rate and catalytic rate are invariant, and the binding rate of topoisomerase changes when DNA supercoiling density *σ* changes.

The workflow is as follows: we first convert the image of ensemble DNA relaxation assay to the intensity of bands using ImageJ. The bands on the DNA relaxation assay show how DNA supercoiling density changes overtime after a specific kind of topoisomerase is added into a supercoiled DNA. We assume that the intensity of bands has a linear relationship with the average supercoiling density. We then infer the supercoiling density at each time point from the intensity data, which is further fitted with an ODE that characterizes the activity of topoisomerase.

For Gyrase, we assumed that its binding rate follows a sigmoidal function of *σ*. From **S17 Fig**, we can see that the sigmoidal curve fits the experimental data from Ashley *et al.* [3] well. Since the amplitude (*k*_1_) of this sigmoidal curve is dependent on the specific concentration of Gyrase used in the experiment, we only used *k*_2_ and *k*_3_ to represent the shape of the curve. In our study, *k*_1_ is inferred from the *in vivo* experiments [6], which has been discussed in Methods. For Topo I, we assumed that its binding rate follows a stepwise function of *σ*: when DNA is negatively supercoiled, the binding rate is a constant; when DNA is relaxed or positively supercoiled, the binding rate is zero. It fits the experimental data from Chong *et al.* [4] well (**S18 Fig**).


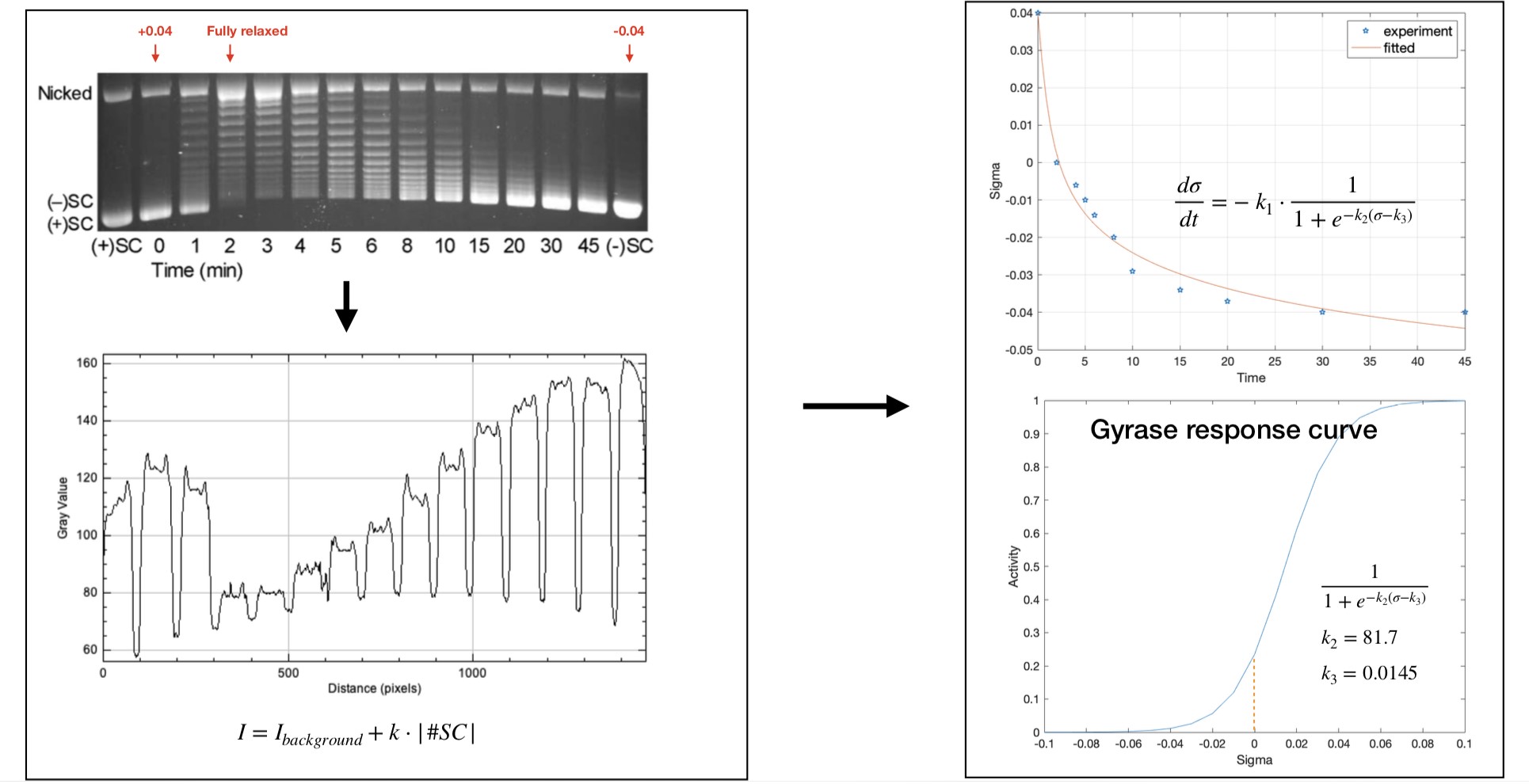


**S17 Fig. Calibration of Gyrase’s response to supercoiling.** Left panel: conversion from gel image to band intensity. Right panel: fitting the supercoiling density data with a sigmoidal curve that represents the Gyrase activity.


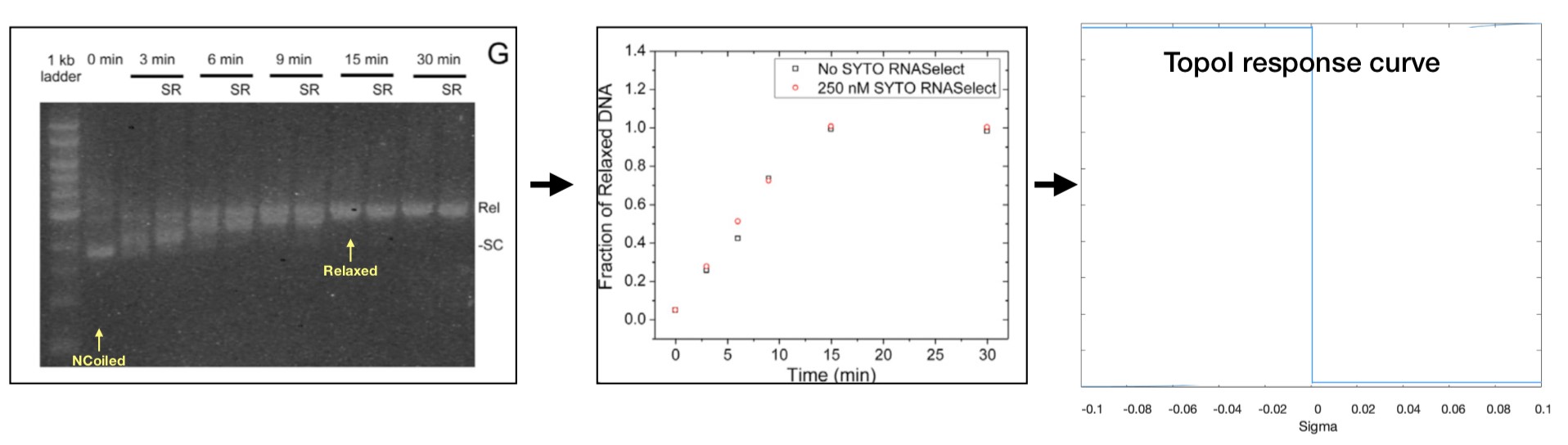


**S18 Fig.** **Calibration of Topo I’s response to supercoiling.** Left panel: conversion from gel image to band intensity. Right panel: fitting the supercoiling density data with a stepwise function that represents the Topo I activity. (Before relaxation, the changes in supercoiling density over time is linear, indicating that the Topo I activity is constant.)

## 3. Parameters used in calculating torques

We adopted Marko [7]’s formulation to calculate the torque generated by the DNA held with a constant force of *F*:


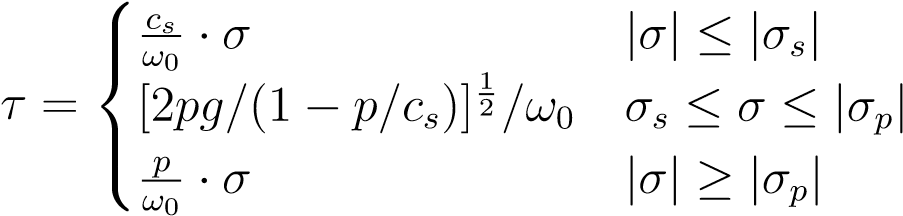


When the absolute supercoiling density is smaller than critical value *σ_s_*, only twists exist, and the torque scales linearly with the supercoiling density. When both twists and writhes exist, the torque is independent of the supercoiling density and only depends on the force. When the absolute supercoiling density is greater than critical value *σ_p_*, only writhes exist, and the torque scales linearly with the supercoiling density. The coefficients *c_s_*, *p*, *g* and critical values *σ_s_*, *σ_p_* can be calculated from mechanical properties of DNA:


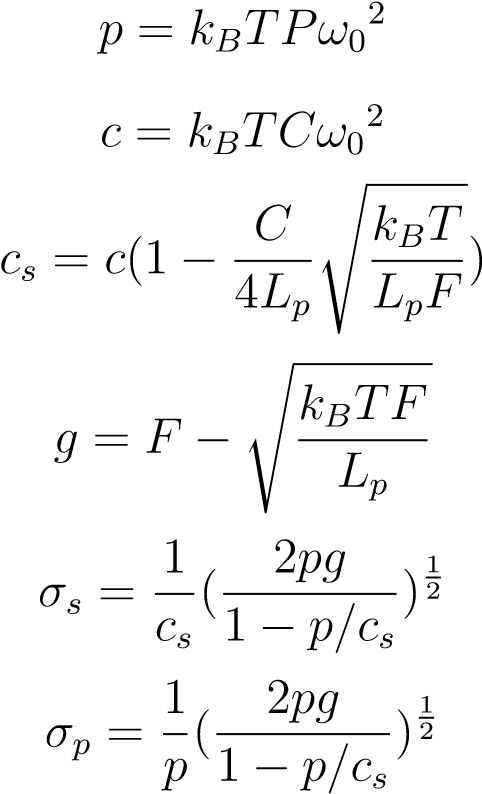


Here we choose temperature (*T*) as 298 *K*, twist persistence length (*C*) as 95 *nm*, stiffness in supercoiled DNA (*P*) as 24 *nm*, DNA bending persistence length (*L_p_*) as 50 *nm*, the contour-length rate of rotation of the relaxed double helix (*ω*_0_) as 2*π/*(3*.*6*nm*) = 1*.*76 *nm*^−1^, and the stretching force (*F*) as 0.15 *pN*.

## 4. Estimating transcription initiation rate from Kim *et al.*’s experimental data

Kim *et al.* [8] measured the elongation rates of RNAP in *lacZ* gene under different levels of IPTG induction. However, the initiation rates were not explicitly measured. Here we aim to deduce the transcription initiation rate from other measurements. Our main assumption is that, at steady state, the mRNA copy number is equal the transcription initiation rate over the mRNA degradation rate. Therefore, the transcription initiation rate could be calculated from the product of the steady-state mRNA copy number and the mRNA degradation rate. Assuming there is on average one genome in the cell. The mRNA number per cell is equivalent to mRNA number per promoter. The measured mRNA lifetime by Kim *et al.* [8] is about 1.5 min, corresponding to a degradation rate of ~0.01/s. For promoter under minimal induction (0.02 mM IPTG), the steady-state mRNA copy number is about 0.2 (Fig 4C in [8]), corresponding to an initiation rate of 0.002 /s. For promoter under 0.05 mM IPTG induction (Fig 1E in [8]), the steady-state mRNA copy number is about 2, corresponding to an initiation rate of 0.02/s. For promoter under 0.2 mM IPTG induction, the steady state mRNA copy number is about 5 (Fig 1E in [8]), corresponding to an initiation rate of 0.05/s.

## References

1. Baker NM, Weigand S, Maar-Mathias S, Mondragon A. Solution structures of DNA-bound gyrase. Nucleic acids research. 2011 Jan 1;39(2):755-66.
2. Krummel B, Chamberlin MJ. RNA chain initiation by Escherichia coli RNA polymerase. Structural transitions of the enzyme in early ternary complexes. Biochemistry. 1989 Sep 1;28(19):7829-42.
3. Ashley RE, Dittmore A, McPherson SA, Turnbough Jr CL, Neuman KC, Osheroff N. Activities of gyrase and topoisomerase IV on positively supercoiled DNA. Nucleic acids research. 2017 Sep 19;45(16):9611-24.
4. Chong S, Chen C, Ge H, Xie XS. Mechanism of transcriptional bursting in bacteria. Cell. 2014 Jul 17;158(2):314-26.
5. Nöllmann M, Stone MD, Bryant Z, Gore J, Crisona NJ, Hong SC, Mitelheiser S, Maxwell A, Bustamante C, Cozzarelli NR. Multiple modes of Escherichia coli DNA gyrase activity revealed by force and torque. Nature structural & molecular biology. 2007 Apr;14(4):264-71.
6. Stracy M, Wollman AJ, Kaja E, Gapinski J, Lee JE, Leek VA, McKie SJ, Mitchenall LA, Maxwell A, Sherratt DJ, Leake MC. Single-molecule imaging of DNA gyrase activity in living Escherichia coli. Nucleic acids research. 2019 Jan 10;47(1):210-20.
7. Marko JF. Torque and dynamics of linking number relaxation in stretched supercoiled DNA. Physical Review E. 2007 Aug 29;76(2):021926.
8. Kim S, Beltran B, Irnov I, Jacobs-Wagner C. Long-distance cooperative and antagonistic RNA polymerase dynamics via DNA supercoiling. Cell. 2019 Sep 19;179(1):106-19.
