# Supplementary material for: A spatially resolved stochastic model reveals the role of supercoiling in transcription regulation
Source: PLoS Comput Biol. 2022 Sep 19;18(9):e1009788. doi: 10.1371/journal.pcbi.1009788 (PMC9522292; doi:10.1371/journal.pcbi.1009788)
Supplement: Attachment — Submitted filename: Response_letter_Yuncong_0526.docx [file pcbi.1009788.s021.docx]

**We thank the reviewers for their constructive comments. Below we provide point-to-point responses (blue) to reviewers’ comments (black).**

Reviewer #1: In general, this is a good paper which addresses an important and timely issue related to the role of supercoiling in modulating transcriptional dynamics. The authors have mostly done a good job in describing their ideas and in showing how they lead to a variety of interesting biological findings, some of which help explain existing data.

We thank the reviewer’s positive affirmation of our work.

I do however have a number of relatively modest requests for clarification of several points in order to make this work easier to understand and easier to place in the context of a number of other very recent papers on the same topic.

• The authors have made understanding some aspects of their model more difficult than necessary. In my opinion, they should move the torque model presentation from the SI to the main text and they should state much more clearly than is now the case that RNAP velocity is determined by torque difference.

We have moved the torque model from the SI to the main text (in section “**Modeling supercoiling-sensitive transcription elongation**”). We also described in more detail how elongation is modulated by torque difference in the same section. Specifically, we didn’t impose the RNAP velocity to be torque-dependent. Instead, we only allowed the stall propensity to be torque-dependent.

====================

• The authors should contrast the treatment of RNAP rotation here with that of other works in the literature. In particular, several papers have pointed out that the moment of inertia for RNAP and hence its rotational drag will increase as a result of RNA molecule growth and ribosome attraction, as the transcription process moves forward. The authors have not included this effect which may or may not be important; this issue should be discussed in the paper.

We have now included more discussions in the revised manuscript. See lines 222 to 234 and copied below:

*“When the length-dependent rotational drag of RNAP is considered, the RNAP would be harder to rotate as the transcript becomes longer, and more complicated behavior might be yielded. If the RNAP rotation is the primary pathway to release the inter-RNAP torsional stress, for very strong promoters, the elongation rate will be limited by the rotation velocity, and the elongation rate could decrease as the initiation increases. This phenomenon will be more pronounced in longer genes. The non-monotonic variation of elongation rate with promoter strength and its gene length dependence is a unique behavior of the length-dependent rotational drag model [26]. Although our model cannot make that kind of prediction, it is a good approximation when RNAP rotation is not the dominant pathway for inter-RNAP torsional stress release. Our model shows that as long as the RNAP molecules rotate slowly enough (i.e., the rotational drag is large enough), the cooperative behavior between RNAP molecules holds and is not sensitive to the specific rotation rate (****S9 Fig****), which qualitatively agrees with the arguments in [28].”*

• The discussion of supercoiling diffusion is quite confusing. In fact, the authors use completely biased motion down the gradient which is hardly what one usually means by diffusion. I am not exactly sure how fixing this rate, which is more of a drag coefficient, can be compared to measurements of a diffusion rate; this point needs clarification.

We apologize for not explaining this point more clearly. In our original submission, we did not model explicitly random, unbiased diffusion of supercoils in order to alleviate the computational cost. Note that under this scenario, we could still use the same rate constant in the explicit random walk (k = 2D/L^2, where L is the length of the DNA segment and D is the diffusion coefficient) as the drift parameter in the biased random walk.

In the revised submission, we now have added unbiased, explicit random walk (**S13 Fig**). We tested different diffusion coefficients and observed similar model behaviors as that in the biased random walk model.

====================

• the statement on line 402, “we propose that intergenic supercoiling dynamics may mediate communication between neighboring genes” with no references given suggest that this is a new hypothesis. This should be corrected as it is not true.

Thanks for catching this oversight. We have now added the corresponding references.

Also, as far as I can recall, the case of divergent promoters was studied by Kim et al and gave rise to lower transcription, in disagreement with the initiation-dominated result given here. Theoretical models do not have to agree with all aspects of the data in order to be interesting and useful, but disagreements should at least be discussed

We found a mistake in the analysis of the divergent promoters in our previous code. We have updated our results in **Fig 6**. The new result suggests that both the overall mRNA production and the elongation rate of the reporter gene decreases with the increased promoter strength of the neighboring gene, which is consistent with **Fig 6** of *Kim et al., 2019.*

• Finally, the authors are missing any mention of a new paper on this subject, “Collective polymerase dynamics …. “ by Sevier and Hornoz, bioarxiv (2021)

Thanks for pointing out. We have now added this reference.

====================

Reviewer #2: The work here presents a comprehensive quantitative model on supercoiling regulation in RNA polymerase (RNAP) transcription, incorporating the RNAP initiation, elongation, DNA supercoiling dynamics/diffusion, topoisomerase activities, and topological domain formation. The model is then implemented in biological context of multiple gene transcriptions with convergent, divergent, and co-directional multi-RNAP configurations, which lead to differently regulated collective behaviors of RNAPs for enhanced or inhibited productivities, due to the translocation-induced supercoiling and intergenic communication.

The model is essential in technically integrating RNAP transcription, supercoiling dynamics and topoisomerase activities altogether, providing a reasonable nice description of the overall process of supercoiling regulated transcription.

We thank the reviewer’s positive affirmation of our work.

Nevertheless, physical clarifications in the model construction appear to be lack of, in particular, on explicitly elucidating time scales of various sub-processes, which make the physical interpretations of current model not highly convincing. The model assumptions are not made sufficiently clearly in the beginning. In addition, as a quantitative model, various values of the parameter sets (e.g. different supercoiling diffusion rates, different topoisomerases) have not been tested, stability and/or robustness of the model or conclusions has not been well addressed. Therefore, the above major issues need to be resolved before this work being considered for publication.

We apologize for not making the model description sufficiently clear. We have added an explicit description of model assumption in the beginning of “**Methods**”, and a table for the timescale of the major subprocesses in the model (**Table 1**). We’ve also varied several model parameters (supercoiling diffusion coefficients, and TopoI unbinding rate) and tested the robustness of the model.

Specific points for the authors’ consideration of improvements are listed below:

1) Page 7 line 65-67 on Kim et al [13]: how differently the RNAP initiation speeds vary under supercoiling comparing to without supercoiling? What is the measured promoter strength (e.g. how many RNAPs initiate per seconds)?

Under optimal supercoiling density (-0.06), the initiation rates will be able to reach the k_max. The measured initiation rate will be different from k_max due to supercoiling. We summarize the measured initiation rate under different k_max in **S4 Table**.

====================

2) Figure 1 caption: several claims are made, such as “(A) a negatively supercoiled promoter is favored over a positively supercoiled promoter for RNAP binding and open complex formation” without references: pls either cite related literature or refer to specific text in current ms for further interpretations (and citations). Note that (B) for twin-domain supercoiling and (D,E) on Topo I and Gyrase all bear such issues (lack of references). Pls check similar lack of references for text elsewhere as well (e.g.,line 129-130 on RNAP footprint of 32 bp and Gyrase binding site 137 bp).

Thanks for pointing out these oversights. We have now added these references.

====================

3) Modeling details: e.g. line 131-132 “to describe the spatial distribution of RNAP, topoisomerases, and supercoils at different positions along the DNA over time”: where are Topo I and Gyrase placed (upstream and downstream), and where are supercoiling (negative and positive) produced, and how fast they are generated (supercoiling generation) and persist (supercoiling relaxation), and how fast the supercoiling diffuses, with references, parameter values (not only provided in supplementary table) and justifications on the ranges.

The TopoI and Gyrase are placed randomly (indiscriminately) on the unoccupied DNA. They are not forced to be in the upstream or downstream RNAP. The response of their catalysis activity to the supercoiling density of the bound segment is then parameterized based on previous studies (see **SI**). This placement is stated in the “**Parameterizing the binding kinetics and catalytic activities of topoisomerases**” section.

The initialization of supercoiling is described in “**modeling diffusion of supercoiling”** section. The production of supercoiling by transcription is described in “modeling the effects of transcription on supercoiling” section. We have also made a table (**Table 1**) to show the parameter values and corresponding time scales.

====================

4) Time scales and modeling assumptions in general: Line 136-138 “To describe the dynamics in the coupling of transcription and supercoiling, we focused on two questions: (1) how DNA supercoils are generated and dissipated by transcription and other processes, and (2) how transcription responds to DNA supercoiling”. The above questions are well raised, but poorly addressed/summarized in the model construction here. This is the right place to put up explicitly the **time scales** for supercoiling generation, dissipation, diffusion, and how fast the two typical types (?) of topoisomerases bind and unbind, which time scales are fast, which are slow, which are certain/ determined already, and which are uncertain and can be possibly tuned etc.. Meanwhile, it is the right place to list a **summary of assumptions explicitly**, though several assumptions spread around followed text.

We have added an overview of the model with a summary of assumptions, in the beginning of “**Methods**”. We have also added a table to show the time scale of the major processes that contribute to supercoiling generation, dissipation, and diffusion (**Table 1**).

====================

5) Modeling Diffusion of Supercoiling and assumption: As the authors indicate (line 150-151) that twists and writhes diffuse differently along DNA. Even the writhes can also fast hop over a long distance (e.g. on ~1 m/s from van Loenhout Science 2012). The authors assume that torsional stress diffuses relatively slowly, so only taking writhe diffusion because DNA exists mostly in the form of writhes under physiological conditions. This assumption surely leads to supercoiling density accumulation between adjacent RNAPs (FIG 2E) and two neighboring genes for codirectional case (FIG 4C). If assuming that the torsional stress diffuses as twists, which is much faster, between codirectional RNAPs and two genes, the positive and negative supercoiling may always cancel out fast. The authors only consider one limiting case for the torsional stress propagation. Right? If so, the authors should emphases this when drawing some conclusions (e.g., some conclusions related to FIG 2 and FIG 4.). Different assumptions lead to different conclusions.

Thanks for raising these possibilities. We have now performed additional simulations testing various diffusion coefficients (from 0.002 µm^2^/s to 2 µm^^2^/s). The results are summarized in **Fig 3**. The observation is that indeed at high supercoiling diffusion (D=2 µm^^2^/s), the cooperation disappears.

====================

6) Modeling diffusion as a biased random walk (line 166-167) to reduce computation cost: the authors should be cautious about this practice as free energy is introduced in the biased walk (as for molecular motor) such that the system operates in non-equilibrium; some of conclusions in regard to interaction of RNAPs with supercoiling may be impacted and should be re-examined.

This point was also raised by Reviewer #1. We have now included new simulations with explicit unbiased random walk for various supercoiling diffusion coefficients (from 0.002 um^2/s to 0.2 um^2/s) and the results are summarized in **S13 Fig**. In essence, we observed similar model behaviors as that in the biased random walk model.

====================

7) Modeling topoisomerase binding/unbinding: it makes more sense to assume binding or on rate of proteins the same (i.e. diffusion limited), while dissociation or off rates vary as protein affinities with DNA differ. Besides, the binding/unbinding kinetic rates here should be tested in a range of values (compatible with experimental measurements or in a range reasonable in theory) to examine whether different values impact significantly on the RNAP-supercoiling dynamics or even lead to quite different working scenarios.

Thanks for pointing that out. We have performed additional simulations testing various TopoI unbinding rates (from 0.001 /s to 100 /s) and the results are summarized in **Fig 3**.

====================

8) Modeling RNAP counter-rotation (line 214-215): Is the RNAP counter-rotation the only scenario that interprets Chong et al [8]’s measurements? What is the leaky diffusion of supercoil?

RNAP counter-rotation is not the only scenario that interprets Chong et al’s measurements.

The alternative scenario is the premature dissociation of RNAP. However, the premature dissociation contradicts with the current knowledge about the stability of the RNAP-DNA elongation complex. Therefore, we didn’t model this scenario in our model.

We modeled the counter-rotation of RNAP through the diffusion of supercoiling. The detailed reaction is added in section “**Modeling the effects of transcription on supercoiling**”.

====================

9) Results: Line 215-216, in Kim et al [13]: for promoter off case: if the last RNAP has no newly loaded RNAP (due to promoter off) to cancel its negative supercoiling upstream/behind, how could that reduce the forward elongation rate of RNAP? Are the modeling results always in agreement with the Kim et al [13]’s measurements and interpretations?

If the last RNAP has no newly loaded RNAP to cancel its negative supercoiling upstream, the upstream torque will be more negative, and the overall torque will increase, which makes the RNAP more likely to stall, and reduces the elongation rate.

The modeling results don’t always **quantitatively** agree with Kim at al’s measurements. One disagreement is that we cannot reproduce the dramatic reduction in elongation after promoter off (for the group of RNAPs, our elongation rate only reduces from 32 bp/s to 26 bp/s, see **S7 Fig**). Another disagreement is that the elongation rate of the solo RNAP also reduces. We have briefly mentioned this in **Results**.

10) What is the elongation rate for a single RNAP in the same modeling context? How about the elongation rates vs the number of RNAPs loaded or in co-transcription case? The authors can show such plots for cooperative and antagonistic cases, respectively, or upon different initiation rates.

We have added a figure (**S5 Fig**) to show the elongation rate versus the number of co-transcribing RNAPs. We also showed the elongation rate for a single RNAP and a group of RNAP before and after promoter off (antagonistic case) in **S7 Fig**.

====================

11) Results on cooperativity (line 295-297): why it is not the case that the new RNAP adapts to the slower rate of front RNAP (but font RNAP is pushed faster)? What parameters could directly impact on this cooperativity (or not)?

Before a new RNAP is loaded, the front RNAP has negative supercoiling behind, slowing down the elongation rate. When a new RNAP comes in, the positive supercoiling it generates at its front will cancel some of the negative supercoiling behind the front RNAP, hence allowing it to face less torque. Therefore, the front RNAP will be pushed faster.

We have systematically tested the effects of supercoiling diffusion coefficient and TopoI unbinding rate and tested local RNAP rotation rate on the cooperativity. All these three parameters affect cooperativity. The detailed discussion is in the second part of the Result section “**The cooperative behavior requires fast TopoI unbinding and moderate supercoiling diffusion rates**”.

====================

12) Results on “increased the Topo I activities” (line 304): are the binding or/and catalytic rates increased?

We have removed this part from our current version, and we now modulate the TopoI unbinding rate in the current version. (In the previous version, we referred to the binding rate rather than the catalytic rate.)

====================

13) Figure 2D: what is the DNA supercoiling density for processive/paused RNAP: the supercoiling density on the DNA around RNAP? Why upstream can still have significant positive supercoiling density, while downstream can have significant negative supercoiling density (large initiation rate)?

The supercoiling density for RNAP is defined as the supercoiling density of the DNA segment immediately upstream or downstream of the RNAP molecule. If RNAP occupies segment k, then the supercoiling density for segment k-1 and k+1 will be calculated. The calculation was described in **Methods** and copied below:

*“Supercoiling density is defined as  (Lk-Lk_0_)/Lk_0_, where Lk is the actual number of turns in the DNA segment, and Lk_0_ is the number of turns in the DNA when it is fully relaxed (one turn per 10.5 bp).”*

The processive and paused RNAP are denoted as different species in the simulation, so we can distinguish their supercoiling density.

For large initiation rates, adjacent RNAP molecules could be very close to each other, and the supercoiling density in the inter-RNAP region will be subject to large stochastic noises from the motion of the flanking RNAP molecules. If the leading RNAP molecule happens to run faster than the trailing one, it will generate more negative supercoiling than the positive supercoiling generated by the trailing RNAP molecule, and hence reducing the torque even though the absolute amount of supercoiling density (positive and negative) immediately downstream is high. If the leading RNAP molecule runs slower than the trailing one, the same logic applies.

====================

14) Results on the mRNA production bursty on dynamical looping (line 362): does the number of RNAPs vary significantly? Does that contribute to the mRNA production busty or increased Fano factor? How exactly the dynamical looping was set in the simulation?

We have examined the distribution of number of RNAPs and their elongation rates for a strong promoter. They display much larger variability in the looped case than in the unlooped case, suggesting that the elongation dynamics also contribute the transcriptional bursting. We’ve added a section “**Modeling looping and unlooping**” in Methods to describe how the dynamical looping was set.

====================

Reviewer #3: In this work, Gent et al. developed a comprehensive mathematical model to understand the roles of supercoiling in transcription regulation. They considered many regulations, including RNAP-supercoiling interactions, topoisomerase activities, stochastic topological domain formation, and supercoiling diffusion in all transcription stages. The model setup is very solid, with all the simplification, parameterization, and assumptions well justified by published data. Some of the predictions are very interesting. For example, the cooperative behavior of co-transcribing RNAP molecules could be a universal feature in E. coli gene transcription. Overall, the theoretical analysis in this work is very solid, and the predictions derived from the simulation are worthy of being tested experimentally.

We thank the reviewer’s positive affirmation of our work.

Major.

1. For the Modeling supercoiling-sensitive transcription initiation section, is there any difference or revision of the equations compared to ref. 21? If not, please clearly state it. If yes, please clearly state the revision and difference.

Yes, there’s a difference. The difference is that we impose an upper bound (k_max) and a lower bound (k_min) on the initiation rate; while in ref.21, the initiation rate is not bounded. i.e., we implemented a truncated version of the model in ref.21. We’ve clarified it in the main text.

====================

2. The discussion of the supercoiling-dependent models in the design of synthetic gene circuits is very important and interesting. The simple two-gene system could yield complex behaviors due to the supercoiling dynamics. The authors could further expand this by discussing the hidden interaction between circuits and host cells (PMCID: PMC7870843, PMC7246135). For example, the resource competition for the ribosome and RNPA could lead to additional noise into the system but also constrain the noise level compared to the unlimited resource case (Advanced Genetics, 2022, 2100047). This is highly related to the additional transcription noise introduced in Fig 3C&F.

Thanks for suggesting those papers. They make an interesting conclusion that the competition for RNAP and ribosome could bring additional effects on the translational noise. However, currently we are uncertain how these works are related to our study. The observed effect is mostly pronounced in high-copy genes (in synthetic circuit, where multiple genes are expressed on high copy plasmid) but could be negligible for low-copy genes (like chromosomal genes), which is the case for our study. Furthermore, while we may miss extra sources of noise due to the competition in our model, this noise would be considered as extrinsic noise and hence not applicable for our system, as our model focuses on intrinsic noise, which is the birth and death of individual transcripts due to the action of RNAP, DNA and supercoiling.

====================

Minor.

3. Page 7, line 63, examples with citations are needed to clarify the emergent properties in transcription.

Sorry that we did not make it clear. The emergent behavior explicitly refers to the subsequent three paragraphs (i.e., the collective behavior of RNAP at the single gene level, the communication between two genes at the multi-gene level, and the transcriptional bursting at the domain level.)

====================

4. Citations are needed in line 99, page 9.

Thanks for pointing out. We’ve added the corresponding references.

====================

5. page 17, which figure is Fig. SX?

This is a typo. We’ve corrected it in the current version.

====================

6. Refer fig. S12-13 in the main text as well. Is Fig. S11 missing?

We’ve corrected the order and reference of supplementary figures in the current version.

====================

7. Acknowledge the funding from National Science Foundation.

PLOS doesn’t allow mentioning of funding information in Acknowledgement. We have provided the information in a separate financial disclosure statement.
