# Supplementary material for: A spatially resolved stochastic model reveals the role of supercoiling in transcription regulation
Source: PLoS Comput Biol. 2022 Sep 19;18(9):e1009788. doi: 10.1371/journal.pcbi.1009788 (PMC9522292; doi:10.1371/journal.pcbi.1009788)
Supplement: Attachment — Submitted filename: Response_letter_Yuncong_0809.docx [file pcbi.1009788.s022.docx]

**We thank the reviewers for their constructive comments. Below we provide point-to-point responses (blue) to reviewers’ comments (black). To facilitate the reading of the revised manuscript, we highlighted major changes in blue in both the main text and supplemental information. Small typos and grammar corrections are not highlighted.**

Reviewer #2: The revisions provide by the authors are substantial, and the overall work reads much more impressive than the first submission. The model is highly comprehensive and presented in well balance now. Most of my concerns have been addressed. There are some additional issues, however, that I recommend for the authors’ consideration to further clarify or comment:

We thank the reviewer’s positive affirmation of our work.

1) The authors emphasize RNAP stall in Discussion (page 28), which has been suggested by Ma et al [ref10] from in vitro single molecule experiment. In Chong et al [ref 8], the initiation speed can be reduced approaching zero, while the elongation speed reduces but to about e.g. 1/3, or say, no stall appears for elongating RNAPs. Is there any scenario that explains such behaviors based on current model?

We believe that the difference is due to the different experimental conditions used in Chong *et al*. In the work by Chong et al., the elongation rate was measured both *in vitro* and *in vivo*.

In the *in vitro* setup of Chong *et al.*, one end of the linear DNA is tethered to the surface, and the other end of the DNA is free to rotate. Therefore, torsional stress generated by transcription could be dissipated by DNA rotation. In our work, we modeled chromosomal DNA in which free DNA rotation is not allowed, and hence not expected to recapitulate the *in vitro* behavior.

For the *in vivo* case, the transcriptional activity used to measure the elongation rate is sampled every 2 min (Fig 6 in Chong *et al*). In our model, each stall typically lasts for seconds (**S8 Fig**); therefore, the stall events will not be captured in the low-time-resolution measurement as that in Chong *et al*.

2) For the diffusion and twist: the authors modeled only the writhe diffusion (as the supercoiling diffusion), how about the twist? Does the twist diffuse differently? The supercoiling diffusion has been measured experimentally (van Loenhout et al ref [29]). In a recent modeling work on two-phase dynamics of supercoiling (Wan & Yu, Biophy J 2022), the diffusion of supercoiling is characterized via the boundary of the stretched phase (twist) and plectoneme (writhe), which may support effectively current treatment of diffusion. Meanwhile, the twist-writhe should together contribute to the toque, which current model does not seem to incorporate such level of physics details to calculate the torque.

In our current model, the twist does contribute to the torque: we adopted the framework from Marko to calculate the torque directly from the supercoiling density, regardless of twisted or writhed state (line 264 - 269). However, when modeling diffusion, we didn’t differentiate the twist and writhe, and used a single diffusion coefficient for the transport of “linking number” for the ease of the computation. We discussed this issue in p. 30 (line 639 - 648) and copied the text below:

*“We also point out that our model simplified the complex dynamics of supercoiling, i.e., the different diffusion behavior of the twist and the writhe [63] and the hopping of plectonemes [29]. According to a recent two-phase dynamic model of supercoiling [63], twist diffusion reaches fast equilibrium, compared to the RNAP translocation, while writhe diffusion is slow. Meanwhile, the transition from the twisted to the writhed state could occur dynamically during the translocation process [63]. Our model did not differentiate the twisted or writhed states and used a single diffusion coefficient to describe the transport of the linking number for the ease of computation. Furthermore, according to van Loenhout et al. [29], plectonemes could suddenly disappear and reappear at thousands of base pairs away on the DNA, known as "hopping." The hopping behavior does not rely on diffusion. It is worth investigating how those complex behaviors affect transcription with a more refined physical model.”*

3) In this model, it seems that the steady state behaviors are described, but how about the dynamic process of supercoiling generation (or relaxation or dissipation), e.g., how the supercoiling density builds up as the RNAP elongates?

The model assumes a steady-state behavior in the sense that the number of RNAP molecules on the DNA does not fluctuate significantly due to the continuous loading and exit of RNAP molecules at the promoter and terminator respectively. We model the dynamic process of supercoiling generation and dissipation explicitly as described in section “*Modeling the effects of transcription on supercoiling*”. Additionally, in Fig 2E we provided a good visualization of how the supercoiling density builds up as RNAP elongates.

Does the supercoiling grow continuously, or discretely? In a previous model (Jing et al 2018 Phys Biol 15, 066007), it was assumed that the level of supercoiling builds up one-by-one (discretized) for every RNAP elongation such that multiple RNAP runs lead to multiple kinetic processes of supercoiling generation. Does the supercoiling dynamics demonstrated in current model suggest similar or different ideas?

In our model, each displacement of RNAP will change the local supercoiling profile, which is further altered by diffusion and topoisomerase actions.

Our treatment is different from the previous model (Jing et al 2018 Phys Biol 15, 066007). In Jing *et al*’s model, the amount of supercoiling induced is determined by the length of the transcript. In our model, the amount of supercoiling induced is affected by the profile of local topological barriers (for example, RNAP molecules) and local supercoiling. For example, assuming that a RNAP molecule is 300 bp from a downstream RNAP molecule, and there are 30 twists/writhes in between; this scenario leads to a supercoiling density of (30-(300/10.5))/(300/10.5) = 0.05. If this RNAP goes one segment further (60 bp) and its downstream RNAP remains still, the inter-RNAP distance will become 240 bp, and the supercoiling density will become (30-(240/10.5))/(240/10.5) ~= 0.315.

Besides, the authors do not show elongation rates for individual RNAPs, which if shown as a distribution would be nice to reflect the fluctuation behaviors of the convoy of RNAP.

Thanks for the suggestion. We have added a figure (**S6 Fig**) for the distribution of elongation rate under different initiation rates. The major observation is that the higher the initiation rate, the smaller the fluctuations.

In addition, in Fig 2D middle right, why the downstream supercoiling density is mainly negative for the processive RNAP (green ones)? Should the downstream supercoiling density to be dominantly positive for the transcribing RNAP?

It is true that every movement of RNAP molecule generates positive supercoiling downstream, but in a convoy of RNAP molecules, those positive supercoiling can be easily cancelled out by the negative supercoiling generated by the downstream RNAP molecule. The negative downstream supercoiling we observed here can be explained by the excessive movement of its immediate downstream RNAP molecule.
